# AARS1‐Mediated H3K27 Lactylation Rewires Glycolysis to Sustain Aggressive and Recurrent Bladder Cancer

**DOI:** 10.1002/advs.77479

**Published:** 2026-08-30

**Authors:** Qin Yuan, Tianbao Song, Yipeng He, Sihan Xia, Yaoqiu Huang, Wenlin He, Qin Yi, Zefeng Wang, Peihan Wang, Chenbo Huang, Linzhi Li, Zhen Yin, Fan Cheng, Weimin Yu

**Affiliations:** ^1^ Department of Urology Renmin Hospital of Wuhan University Wuhan Hubei China; ^2^ Taikang Center for Life and Medical Sciences of Wuhan University Wuhan Hubei China

**Keywords:** AARS1, bladder cancer, eltrombopag, glycolysis, H3K27 lactylation, HK2, recurrence

## Abstract

Recurrence and progression remain major clinical challenges in bladder cancer (BC), yet the mechanisms linking glycolysis to persistent malignant states remain incompletely defined. Here, we identify alanyl tRNA synthetase 1 (AARS1) as a clinically relevant driver of aggressive and recurrent BC. AARS1 is upregulated in BC tissues, enriched in muscle invasive and recurrent tumors, and associated with unfavorable survival. Functionally, AARS1 promotes proliferation, epithelial mesenchymal transition, invasion, apoptosis resistance, tumor growth, and lung colonization. Mechanistically, AARS1 enhances phosphoinositide 3 kinase (PI3K) pathway output, glycolytic flux, and lactate production. Increased lactate availability is linked to histone H3 lysine 27 lactylation (H3K27la) enrichment at the hexokinase 2 (HK2) promoter, increased chromatin accessibility, and HK2 transcriptional activation, supporting an HK2 centered metabolic and epigenetic reinforcement program. HK2 perturbation attenuates AARS1 associated glycolytic and malignant phenotypes. Through structure guided screening and surface plasmon resonance validation, we further identified eltrombopag as an AARS1 binding compound that pharmacologically suppressed the AARS1 associated PI3K, glycolysis, lactate, H3K27la, and HK2 program and restrained tumor progression in preclinical models. These findings define an AARS1 associated metabolic and epigenetic program in BC and nominate AARS1 targeting strategies as a direction for further therapeutic development.

## Graphical abstract

AARS1 drives PI3K–AKT–mTOR‐dependent glycolysis in bladder cancer, promoting lactate accumulation, increased lactylation, H3K27la enrichment at the HK2 promoter, and HK2 transcription. HK2 reinforces glycolysis, sustaining a metabolic–epigenetic program associated with tumor progression, recurrence, and metastasis. Eltrombopag binds AARS1 and suppresses the AARS1‐associated signaling program, highlighting a potential therapeutic vulnerability in bladder cancer.

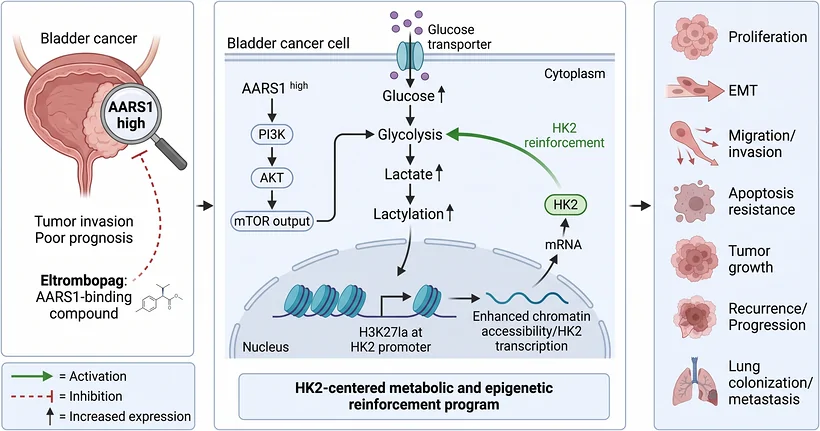

## 1 Introduction

Bladder cancer (BC) remains one of the most prevalent malignancies of the urinary tract and continues to impose a substantial and rising global health burden [1]. Clinically, most patients present with non–muscle‐invasive bladder cancer (NMIBC), which can often be initially controlled by transurethral resection followed by intravesical therapy. However, recurrence remains frequent, and a clinically meaningful fraction of high‐risk NMIBC ultimately progresses to muscle‐invasive bladder cancer (MIBC) [2, 3]. Once invasion and metastasis occur, long‐term outcomes remain unsatisfactory despite rapid advances in immune checkpoint blockade and antibody–drug conjugates [4, 5, 6, 7, 8], underscoring the urgent need to define mechanistically grounded, clinically actionable biomarkers and therapeutic vulnerabilities that enable risk stratification, intervention, and translation.

Metabolic reprogramming is a core hallmark of cancer progression, with aerobic glycolysis and the accumulation of lactate representing a prototypical feature of aggressive tumors [9]. Lactate is no longer thought of as just a byproduct of metabolism. Rather, it is a pleiotropic signaling molecule that influences the tumor microenvironment and tumor cell fitness [10, 11]. Accumulating evidence supports that lactate can promote invasion, metastatic dissemination, immune modulation, and therapy resistance through both cell‐intrinsic and microenvironmental mechanisms [12, 13, 14]. Therefore, clarifying how the glycolysis–lactate axis is rewired by upstream oncogenic drivers and how such rewiring is converted into stable malignant phenotypes—represents a critical step toward understanding BC recurrence and progression.

A major conceptual advance in this area is the emerging link between lactate and chromatin regulation. In 2019, Zhang and colleagues identified histone lysine lactylation as a new epigenetic modification derived from lactate metabolism, providing a direct molecular framework for coupling metabolic state to transcriptional control [15]. Subsequent studies further expanded the landscape of lactylation to non‐histone proteins, suggesting broad roles in transcriptional regulation, stress responses, immune signaling, and tumor biology [16, 17]. However, in BC, it remains insufficiently understood how lactylation is initiated by upstream metabolic switches, how specific histone marks are deployed to remodel transcriptional programs, and whether these processes can be therapeutically exploited in a mechanism‐driven manner.

Aminoacyl‐tRNA synthetases (AARSs) have classically been viewed as housekeeping enzymes essential for protein translation, yet growing evidence indicates that AARSs possess diverse non‐canonical functions that influence proliferation, migration, stress adaptation, immunity, and tumorigenesis [18, 19]. Notably, recent work uncovered an unexpected function for alanyl‐tRNA synthetase 1 (AARS1): AARS1 can act as a lactate sensor and a lactyltransferase, catalyzing lactate activation and promoting global lysine lactylation in tumor contexts [20]. These findings raise an important possibility that an AARS may serve as a previously unrecognized hub linking oncogenic signaling, metabolic output, and epigenetic regulation. Whether AARS1 drives a BC‐specific metabolic–epigenetic circuit, contributes to recurrence‐associated aggressiveness, and can be effectively targeted pharmacologically remains unknown.

Here we identify AARS1 as a recurrence‐ and progression‐associated driver in bladder cancer and delineate a mechanistic cascade in which AARS1 activates PI3K/AKT‐dependent glycolytic lactate production to enforce histone lactylation and sustain transcription of glycolytic effectors through a feed‐forward circuit. Leveraging a drug‐repurposing strategy, we further integrate structure‐guided prediction with biophysical validation to uncover Eltrombopag as a candidate AARS1‐targeting agent and evaluate its capacity to disrupt the AARS1‐driven metabolic–epigenetic program in preclinical models.

## 2 Materials and Methods

### 2.1 Clinical Specimens and Ethics

The WHRM‐BLCA tissue cohort from Wuhan University Renmin Hospital was analyzed, including tumor tissues and matched adjacent non‐tumor tissues with annotated clinicopathological variables and follow‐up information. Written informed consent was obtained from all participants. The study was approved by the Institutional Ethics Committee of WHRM (Approval No. WDRY2025‐K080). In addition, 87 paired fresh tumor and adjacent tissues were collected for immunoblot validation under the same ethical framework. Fresh specimens were snap‐frozen in liquid nitrogen and stored at −80°C until use.

### 2.2 Immunohistochemistry and Quantification

Paraffin sections were deparaffinized and rehydrated, followed by antigen retrieval using heat‐induced epitope retrieval (HIER) in 10 mM sodium citrate buffer (pH 6.0) at 95–100°C for 20 min, and then allowed to cool naturally at room temperature for 20 min. Endogenous peroxidase activity was quenched with 3% H_2_O_2_ for 10 min at room temperature, followed by blocking with 5% BSA for 30 min. Sections were incubated with primary antibodies overnight at 4°C. Detailed antibody information, including supplier, catalog number, host species, application, and working dilution, is provided in Table S1. On the next day, sections were incubated with HRP‐conjugated secondary antibodies and developed with DAB, counterstained with hematoxylin, dehydrated, and mounted. Isotype‐matched IgG was used as a negative control. IHC signals were semi‐quantified using digital pathology analysis in QuPath (v0.3.x). Quantification was performed using percentage of positive area. For TMAs, whole‐core quantification was performed and averaged across multiple cores per case. Cut offs for high vs low expression were defined by the cohort median.

### 2.3 Public Datasets and Bioinformatics

TCGA‐BLCA transcriptomic and clinical data were downloaded from the TCGA portal (access date: 2023.9). GEO datasets (GSE3167) were obtained from the GEO repository. Differential expression analyses were performed in R (v4.3.2). For RNA‐seq count data, differential expression was assessed using DESeq2 (v1.40.2) and limma (v3.56.2) was applied for microarray data. KEGG enrichment was conducted using clusterProfiler (v4.8.3) and GSEA was performed using fgsea (v1.26.0). Survival studies were conducted utilizing Kaplan–Meier estimates alongside log‐rank testing, while hazard ratios (HRs) were computed by Cox proportional hazards regression, as implemented in the survival (v3.5‐7) and survminer (v0.4.9) packages.

### 2.4 Cell Lines and Culture

Bladder cancer cell lines (T24, UMUC3, 5637, J82, and TCCSUP) and normal urothelial cells (SV‐HUC‐1) were purchased from Procell Life Science & Technology Co., Ltd. (China). All cell lines were authenticated by STR profiling and routinely tested for mycoplasma contamination (every 4 weeks). Cells were maintained at 37°C with 5% CO_2_ and cultured in vendor‐recommended basal media as follows: T24 in McCoy's 5A medium; UMUC3, J82, and TCCSUP in MEM; 5637 in RPMI‐1640; and SV‐HUC‐1 in F‐12K. Unless otherwise indicated, media were supplemented with 10% fetal bovine serum (FBS) and 1% penicillin and streptomycin. Cells in logarithmic growth phase were used for experiments, and passage numbers were restricted to P5–P20.

### 2.5 Plasmids, Lentiviral Transduction and Stable Lines

AARS1 overexpression, AARS1 shRNA knockdown, and corresponding control constructs were generated using a lentiviral system. Full‐length AARS1 was cloned into a CMV promoter–driven lentiviral expression vector with a puromycin resistance cassette (Puro). The AARS1‐5A mutant (M46A, R77A, N216A, D239A, and G241A) was generated by site‐directed mutagenesis and verified by Sanger sequencing. AARS1 and HK2 overexpression and knockdown (siRNA and shRNA) were generated and validated in an analogous manner (sequences in Tables S2 and S3). Lentiviral vector construction, virus packaging, and titer determination were performed by GeneChem (Shanghai, China). Target cells were transduced according to the manufacturer's recommended procedures and selected with puromycin to establish stable cell populations. Transduction and expression efficiencies were validated by RT‐qPCR, immunoblotting, and immunofluorescence. For overexpression experiments, stable cell populations with moderate AARS1 expression were selected for downstream assays to avoid artifacts associated with excessive supraphysiologic protein expression. Expression efficiency was validated by RT‐qPCR, immunoblotting, and immunofluorescence before functional experiments.

### 2.6 Reagents and Treatments

Wortmannin, recombinant human IGF‐1, 2‐deoxy‐D‐glucose (2‐DG), sodium lactate, Eltrombopag, and other key reagents were obtained from commercial suppliers. Detailed reagent information, including supplier, catalog number, solvent, stock concentration, final working concentration, treatment duration, and in vivo dosing schedule where applicable, is provided in Table S4. Unless otherwise indicated, vehicle control groups received equal volumes of DMSO or PBS. Working concentrations and treatment durations used in individual experiments are also indicated in the corresponding figure legends.

### 2.7 Immunoblotting, qPCR, and Immunofluorescence

Cells or tissues were lysed in RIPA buffer supplemented with protease and phosphatase inhibitors. Lysates were clarified by centrifugation, and protein concentrations were determined using a BCA assay. Equal amounts of protein were resolved by SDS–PAGE and transferred onto PVDF membranes. After blocking with 5% non‐fat milk or 5% BSA, membranes were incubated with primary antibodies overnight at 4°C. Detailed antibody information, including supplier, catalog number, host species, application‐specific working dilution, and incubation condition, is provided in Table S1. Enhanced chemiluminescence was used to view the membranes after they had been treated with HRP‐conjugated secondary antibodies. ImageJ (v1.53) was used to quantify band intensities, which were then adjusted to β‐actin for whole‐cell lysates and Histone H3 or H4 for histone protein analysis.

For qPCR, total RNA was extracted using TRIzol and reverse‐transcribed into cDNA. Quantitative PCR was performed using SYBR Green chemistry, and relative expression levels were calculated by the 2^−ΔΔCt^ method with ACTB as an internal control. Primer sequences and amplification conditions are provided in Table S5.

For immunofluorescence, cells were fixed with 4% paraformaldehyde, permeabilized with 0.1%–0.3% Triton X‐100, and blocked with 5% BSA. Cells were incubated with primary antibodies followed by fluorophore‐conjugated secondary antibodies, and nuclei were counterstained with DAPI. Images were acquired using a Leica SP8 confocal microscope. Fluorescence signals were quantified using ImageJ (v1.53). For quantification, images were collected using identical acquisition settings, and at least three independent fields per condition were analyzed. The data are provided as the proportion of positive cells, as shown in the figure legends. Primary and secondary antibodies used for immunofluorescence, together with their working dilutions, are listed in Table S1.

### 2.8 Cell Proliferation, Apoptosis, Migration and Invasion Assays

CCK‐8 assay: Cells were seeded into 96‐well plates at 2000 cells per well. At the indicated time points, CCK‐8 reagent was added and absorbance was measured at 450 nm using a microplate reader. Experiments were repeated at least three times independently.

Colony formation assay: 800 cells per well were seeded into 6‐well plates, and the cells were cultivated for 10–14 days until colonies could be seen. ImageJ was used to count the colonies after they had been fixed with 4% paraformaldehyde and dyed with 0.1% crystal violet. At least three separate experiments were conducted, with duplicate wells used for each condition's analysis.

EdU incorporation assay: DNA synthesis was assessed using an EdU kit according to the manufacturer's instructions. Briefly, cells were incubated with EdU at 10 µM for 2 h, fixed, and processed for fluorescence detection. The percentage of EdU‐positive cells was quantified from at least three random fields per sample under identical acquisition settings. Experiments were performed in at least three independent replicates.

Cell‐cycle analysis: Cells were harvested, fixed in 70% ethanol at 4°C overnight, treated with RNase A, and stained with propidium iodide (PI). Cell‐cycle distribution was analyzed by flow cytometry, and data were processed using standard gating strategies.

Apoptosis assays: Apoptosis was evaluated by TUNEL staining and Annexin V/7‐AAD flow cytometry following the manufacturers’ protocols. For flow cytometry, early and late apoptotic fractions were quantified using a consistent gating strategy across experiments.

Transwell migration and Matrigel invasion assays: For migration assays, 5 × 10^4^ cells in serum‐free medium were seeded into the upper chamber of Transwell inserts, with complete medium containing 10% FBS in the lower chamber as a chemoattractant. After 24 h, migrated cells on the underside of the membrane were fixed and stained, and quantified by counting cells from at least five random fields per insert. For invasion assays, inserts were pre‐coated with Matrigel diluted 1:8 in serum‐free medium, and 1 × 10^5^ cells were seeded per insert; invading cells were quantified after 24–36 h using the same procedure.

Wound‐healing assay: Cells were grown to near confluence in 6‐well plates, and a linear scratch was generated using a sterile pipette tip. After washing, images were captured at 0 h and 24 h. Wound closure was quantified as the percentage reduction of wound area using ImageJ.

### 2.9 Seahorse Metabolic Flux Analysis

Extracellular acidification rate (ECAR) and oxygen consumption rate (OCR) were measured using a Seahorse XFe96 Analyzer (Agilent Technologies). For ECAR, a Seahorse Glycolysis Stress Test was performed. Cells were seeded at 1.5 × 10^4^ cells per well (XFe96 cell culture microplate) and assayed according to the manufacturer's instructions with minor optimization. Compounds were injected sequentially to achieve final working concentrations of glucose (10 mM), oligomycin (1.0 µM), and 2‐deoxy‐D‐glucose (2‐DG, 50 mM). Glycolysis and glycolytic capacity were calculated using Seahorse Wave software based on standard definitions. For OCR, a Seahorse Mito Stress Test was performed with sequential injections of oligomycin (1.0 µM), FCCP (1.0 µM), and rotenone/antimycin A (0.5 µM each). Basal respiration, ATP‐linked respiration, maximal respiration, and spare respiratory capacity were calculated following the manufacturer's algorithm. Each condition included ≥6 technical replicate wells, and every experiment was conducted in a minimum of three separate biological duplicates. ECAR/OCR values were normalized to protein content per well (BCA assay) or cell number as indicated in the corresponding figure legends.

### 2.10 Lactate Measurement and U‐^1^ ^3^C_6_‐glucose Tracing

Lactate measurement: Extracellular lactate was quantified from conditioned media using a lactate assay kit from Nanjing Jiancheng Bioengineering Institute (A019‐2‐1, Nanjing, China) according to the manufacturer's instructions. Lactate levels were normalized to total protein content (BCA), as specified in the corresponding figure legends. Each condition was assayed with ≥3 technical replicates and repeated in at least three independent experiments.

U‐^1^ ^3^C_6_‐glucose tracing and LC–MS analysis: For isotope tracing, cells were washed once and switched to glucose‐free basal medium supplemented with U‐^1^ ^3^C_6_‐glucose (10 mM) and 10% dialyzed FBS, followed by incubation for 6 h at 37°C. Metabolism was quenched by rapidly aspirating the medium and adding pre‐chilled 80% methanol (v/v; −80°C). Extracts were collected, clarified by centrifugation at 4°C, dried under vacuum, and reconstituted for LC–MS analysis. Metabolites were separated by HILIC using a ZIC‐pHILIC column and analyzed on a Thermo Q Exactive high‐resolution mass spectrometer operated in full‐scan mode. Lactate M+3 labeling and isotopologue distributions of related metabolites were quantified from extracted ion chromatograms with appropriate mass accuracy tolerance. Natural isotope abundance correction was performed using AccuCor prior to downstream comparisons of isotopologue fractions.

### 2.11 RNA Sequencing and Pathway Analysis

Total RNA was extracted from control and AARS1‐knockdown T24 cells. Samples with an RNA integrity number (RIN ≥ 7.0) were used for library construction. Poly(A)‐enriched libraries were sequenced on an Illumina platform NovaSeq 6000 to generate paired‐end 150‐bp reads (PE150) with a sequencing depth of ∼30–50 million reads per sample. Raw reads were quality‐controlled and aligned to the human reference genome hg38 using STAR (v2.7.x). Gene‐level read counts were generated using featureCounts (Subread, v2.0.x). Differential expression analysis was performed using DESeq2, and differentially expressed genes were defined by |log2 fold change| ≥ 1.0 and FDR < 0.05. Pathway enrichment analysis was conducted using KEGG over‐representation analysis (clusterProfiler), and gene set enric was performed using fgsea on a ranked gene list. KEGG gene sets were obtained from the KEGG pathway (human pathways, https://www.genome.jp/kegg/).

### 2.12 CUT‐Tag‐seq, ATAC‐seq and Integrative Analyses

CUT‐Tag‐seq: CUT‐Tag was performed following a standard CUT‐Tag protocol using pA–Tn5–mediated tagmentation with an anti‐H3K27la antibody, and the antibody source, catalog number, and amount or dilution used per reaction are provided in Table S1. Briefly, 5 × 10^4^–1 × 10^5^ cells per reaction were processed, and libraries were generated after pA–Tn5 tagmentation and PCR amplification (12–14 cycles). Libraries were size‐selected with AMPure XP beads and sequenced on an Illumina platform to obtain paired‐end 150‐bp reads (PE150) at a depth of ∼20–30 million read pairs per sample, with at least three biological replicates per group.

ATAC‐seq: ATAC‐seq libraries were prepared using a standard transposition‐based workflow. In brief, 5 × 10^4^ cells were collected, nuclei were isolated, and tagmentation was performed using Tn5 transposase. Libraries were PCR‐amplified (8–10 cycles, determined by pilot qPCR or minimal‐cycle criteria), purified, and sequenced as PE150 with ∼40–60 million read pairs per sample (≥3 biological replicates per group). Read processing and alignment: Raw reads were quality‐controlled and adapter‐trimmed using fastp (v0.23.x). CUT&Tag and ATAC reads were aligned to the human reference genome hg38 using Bowtie2 (v2.5.x) with standard end‐to‐end settings. BAM files were sorted and indexed with SAMtools (v1.17); PCR duplicates were removed using Picard (v2.27.x). Reads with MAPQ < 30 and mitochondrial reads were excluded for downstream analyses.

Peak calling and quality metrics: Peaks were called using MACS2 (v2.2.7.x). TSS enrichment profiles and heatmaps were generated with deepTools (v3.5.x) using computeMatrix and plotProfile with a typical window of ±3 kb around TSS. Peak annotation and visualization: Genomic annotation of peaks was performed using ChIPseeker (v1.36.x) with hg38 gene annotations. Genome browser tracks (bigWig) were generated (CPM normalization) and visualized in IGV to examine representative loci.

Integrative analysis: Differential peak analysis was performed by quantifying reads in consensus peak sets and applying DESeq2‐based statistics (FDR‐controlled). Peaks were linked to nearby genes using promoter definitions (typically −2 kb to +0.5 kb around TSS) and nearest‐gene annotation. Candidate targets were prioritized by intersecting i) genes associated with differential H3K27la peaks, ii) genes showing differential accessibility (ATAC), and iii) RNA‐seq differentially expressed genes, yielding high‐confidence candidates.

### 2.13 ChIP–qPCR

Cells were cross‐linked with 1% formaldehyde for 10 min at room temperature, followed by quenching with 125 mM glycine. Nuclei were isolated, and chromatin was sonicated to an average fragment size of 200 to 500 bp. Chromatin prepared from approximately 1 to 2 × 10^6^ cells was incubated overnight at 4°C with an anti‐H3K27la antibody or an isotype‐matched IgG control, followed by capture with protein A/G magnetic beads. Detailed antibody information, including supplier, catalog number, application, and antibody amount used for immunoprecipitation, is provided in Table S1. After extensive washing, immune complexes were eluted, and reverse cross‐linking was performed at 65°C overnight. DNA was purified using a standard spin‐column method. Enrichment of H3K27la at the HK2 promoter was quantified by qPCR using primers listed in Table S6. ChIP‐qPCR signals were presented as percent input, as indicated in the figure legends. Each condition included at least three technical qPCR replicates and was repeated in at least three independent biological experiments.

### 2.14 Molecular Docking, Molecular Dynamics (MD) Simulation and SPR

Protein structure preparation and molecular docking: The structure of human AARS1 was obtained from the AlphaFold Protein Structure Database (UniProt: P49588) and prepared by removing waters, adding polar hydrogens, and assigning Gasteiger charges. Eltrombopag and other screened compounds, where indicated was prepared with protonation states appropriate for physiological pH. AutoDock Vina(v1.2.3) was used for molecular docking with a grid box (typically ∼30 × 30 × 30 Å) centered on the predicted ligand‐binding pocket, an exhaustiveness of 16, and 20 output poses per ligand. The top‐ranked poses were further inspected for binPyMOL (v2.5) and Discovery.

Molecular dynamics simulation: The AARS1–Eltrombopag complex was subjected to all‐atom MD simulation using GROMACS (v2022.3). The protein was parameterized with the AMBER99SB‐ILDN force field, and the ligand was parameterized using GAFF2 with AM1‐BCC charges. The system was solvated in TIP3P water with a minimum distance of 10 Å from the protein to the box boundary, neutralized, and supplemented with 0.15 M NaCl. After energy minimization, the system was equilibrated under NVT (100 ps) and NPT (1 ns) ensembles, followed by a 100 ns production ru2 fs time step was used with LINCS constraints; long‐range electrostatics were treated by PME with a 1.0 nm cutoff. Trajectories were analyzed for RMSD,

Surface plasmon resonance (SPR): A Biacore T200 system was used to do SPR measurements (Cytiva). Purified tag‐free recombinant human AARS1 was immobilized on a CM5 sensor chip via standard amine coupling according to the manufacturer's instructions to a target level of approximately 3000–6000 RU, with a reference flow cell prepared in parallel. Binding assays were conducted at 25°C in a commonly used running buffer (HBS‐EP+, 10 mM HEPES, 150 mM NaCl, 3 mM EDTA, 0.05% (v/v) surfactant P20, pH 7.4). Eltrombopag was prepared as a concentration series spanning 0.625–40 µM (serial dilutions) and injected at a flow rate of 30 µL min^−1^ (association 60 s, dissociation 180 s). A constant DMSO percentage was maintained across all samples, and solvent correction was applied where appropriate. Sensorgrams were double‐referenced and globally fitted to a 1:1 Langmuir binding model to derive KD.

### 2.15 Animal Studies and Imaging

All animal experiments were approved by the Institutional Animal Care and Use Committee of Renmin Hospital of Wuhan University (approval No. WDRY20220806B) and were conducted in accordance with relevant guidelines. Mice were randomly assigned to experimental groups, and investigators were blinded to group allocation during tumor measurement and image quantification whenever feasible. Daily animal monitoring was conducted, and institutional protocols were followed to apply humane outcomes, including excessive tumor burden (maximum tumor volume ∼1500 mm^3^ or ulceration), >15%–20% body‐weight loss, or signs of distress (impaired mobility, persistent hunching, or labored breathing), at which point mice were euthanized immediately.

Subcutaneous xenograft model: BALB/c nude mice (4–6 weeks old) were injected subcutaneously in the flank with 2 × 10^6^ cells suspended in 100 µL PBS. Tumor size was measured every 2–3 days using digital calipers, and tumor volume was calculated as V = (L × W^2^)/2. At the endpoint, mice were euthanized; tumors were excised, weighed, photographed, and processed for IHC and molecular analyses.

Experimental lung metastasis model and bioluminescence imaging: Luciferase‐labeled cells were injected via the lateral tail vein at 1 × 10^6^ cells in 100 µL PBS per mouse. For in vivo bioluminescence imaging, mice received intraperitoneal D‐luciferin at 150 mg kg^−1^, and images were acquired 10–15 min after injection using an IVIS system. Total photon flux (photons/s) was quantified using standard ROI analysis. At the endpoint, lungs were harvested for gross nodule counting and hematoxylin–eosin (H&E) staining.

Drug administration: Wortmannin was administered intraperitoneally (i.p.) at 0.6 mg kg^−1^, twice weekly, following a commonly used xenograft schedule for PI3K inhibition. Recombinant human IGF‐1 was delivered via tail‐vein injection at 1 mg kg^−1^ once daily (0.2 mL per mouse per day), consistent with published tumor‐model protocols used to activate IGF‐1 signaling in vivo. Eltrombopag was administered i.p. at 75 mg kg^−1^ every three days (q3d), consistent with reported antitumor dosing schedules in mice. Throughout the trial, body weight and overall health were tracked.

^1^ ^8^F‐FDG PET/CT: Mice were fasted for 6 h before tracer administration. ^1^ ^8^F‐FDG (7.4–11.1 MBq per mouse; 200–300 µCi) was injected via the tail vein, followed by an uptake period of 60 min under standard warming conditions. PET/CT scans were performed under isoflurane anesthesia, and tumor glucose uptake was quantified as SUV (SUVmean) using the standard definition: SUV = tissue activity concentration (MBq/g) / [injected dose (MBq) / body weight (g)].

### 2.16 Statistical Analysis

Data are presented as mean ± s.d., as indicated in the figure legends. For comparisons between two groups, two‐tailed Student's t‐test was used for normally distributed data, whereas the Mann–Whitney U test was applied for non‐normally distributed variables. For comparisons among multiple groups, one‐way or two‐way ANOVA was performed as appropriate, followed by Tukey's post hoc test for all pairwise comparisons. Survival outcomes were analyzed using Kaplan–Meier curves with the log‐rank (Mantel–Cox) test, and hazard ratios were estimated using Cox proportional hazards regression. A *p* value < 0.05 was considered statistically significant. All statistical analyses were conducted using GraphPad Prism (v9.0) and R (v4.3.2). The number of biological replicates (n) and the statistical tests used are specified in the corresponding figure legends.

## 3 Results

### 3.1 AARS1 Is Upregulated in Bladder Cancer and Enriched in Invasive/Recurrent Cases, Predicting Unfavorable Outcomes

**FIGURE 1:**
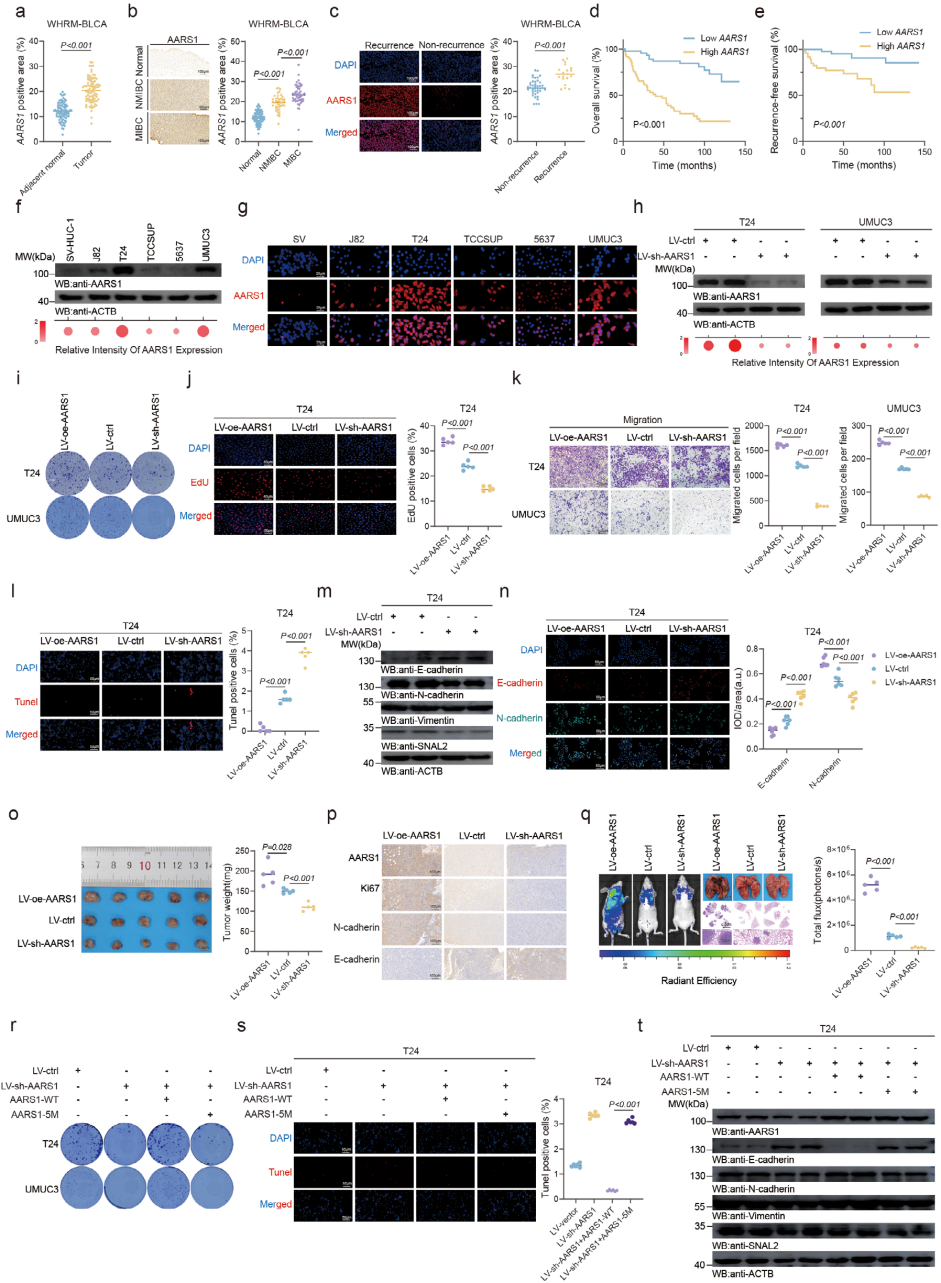
AARS1 is upregulated in bladder cancer and promotes aggressive phenotypes associated with invasion and recurrence. (a) Quantification of AARS1 IHC signal (positive area, %) in adjacent normal versus tumor tissues in the WHRM‐BLCA tissue microarray cohort. Each dot represents one specimen. (b) Representative AARS1 IHC images and quantification of AARS1‐positive area (%) across normal urothelium, non–muscle‐invasive bladder cancer (NMIBC), and muscle‐invasive bladder cancer (MIBC) in the WHRM‐BLCA cohort. (c) Representative immunofluorescence (IF) staining of AARS1 (red) in tumors from non‐recurrent versus recurrent cases in the WHRM‐BLCA cohort (nuclei, DAPI), with quantification of AARS1‐positive area (%). (d–e) Kaplan–Meier analyses of overall survival (d) and recurrence‐free survival (e) in the WHRM‐BLCA cohort stratified by AARS1 expression (high vs low). P values were calculated by the log‐rank test. (f) Immunoblot analysis of AARS1 expression in normal urothelial cells (SV‐HUC‐1) and the indicated bladder cancer cell lines, with densitometric quantification. (g) Representative IF images showing AARS1 protein distribution (red) in SV‐HUC‐1 and bladder cancer cell lines (nuclei, DAPI). (h) Validation of lentiviral AARS1 overexpression (LV‐oe‐AARS1) and knockdown (LV‐sh‐AARS1) in T24 and UMUC3 cells by immunoblotting, with quantification. (i) Colony formation assays in T24 and UMUC3 cells with AARS1 overexpression or knockdown. (j) EdU incorporation assay in T24 cells with the indicated AARS1 manipulation, with quantification of EdU‐positive cells. (k) Transwell migration assays in T24 and UMUC3 cells with the indicated AARS1 manipulation, with quantification of migrated cells per field. (l) TUNEL staining in T24 cells with the indicated AARS1 manipulation, with quantification of TUNEL‐positive cells. (m) Immunoblotting of EMT‐related markers (E‐cadherin, N‐cadherin, Vimentin, and SNAIL2) in T24 cells following AARS1 overexpression or knockdown. (n) IF staining of E‐cadherin (red) and N‐cadherin (green) in T24 cells with the indicated AARS1 manipulation (nuclei, DAPI), with quantification (IOD/area). (o) Representative images of subcutaneous xenograft tumors derived from T24 cells with AARS1 overexpression or knockdown, with endpoint tumor weight quantification. (p) Representative IHC staining of AARS1, Ki67, N‐cadherin, and E‐cadherin in xenograft tumors from the indicated groups. (q) Bioluminescence imaging of lung colonization following tail‐vein injection of luciferase‐labeled T24 cells with the indicated AARS1 manipulation; representative lung images and H&E staining are shown, with quantification of total flux (photons/s). (r) Colony formation assays for rescue experiments in T24 and UMUC3 cells: AARS1 knockdown followed by re‐expression of AARS1‐WT or the catalytically impaired AARS1‐5 M mutant (M46A/R77A/N216A/D239A/G241A), as indicated. (s) TUNEL staining and quantification in T24 rescue settings corresponding to panel (r). (t) Immunoblot validation of AARS1 re‐expression (WT vs 5 M) and EMT marker changes in T24 rescue experiments. Data are presented as mean ± s.e.m. unless otherwise indicated. Two‐group comparisons were analyzed by two‐sided Student's t‐test; multiple‐group comparisons were analyzed by one‐way ANOVA with appropriate multiple‐comparison correction. For cellular assays, images are representative of at least three independent experiments with similar results. Scale bars are indicated in the images.

In the WHRM‐BLCA tissue microarray cohort, immunohistochemistry (IHC) revealed a markedly higher AARS1‐positive area in tumor tissues than in matched adjacent tissues (Figure 1a). AARS1 expression was further increased in MIBC compared with NMIBC (Figure 1b). Clinically, recurrent tumors exhibited significantly higher AARS1 levels than non‐recurrent tumors (Figure 1c). These observations were corroborated by public datasets: AARS1 mRNA was significantly elevated in TCGA‐BLCA tumors versus adjacent tissues and independently validated in the GEO cohort GSE3167 (Figure S1a–c). Consistently, western blotting of freshly collected patient specimens confirmed higher AARS1 protein abundance in tumors than in adjacent tissues, with further elevation in recurrent cases (Figure S1d,e). Kaplan–Meier analyses showed that high AARS1 expression was associated with significantly worse OS, RFS and PFS in the WHRM‐BLCA cohort and public datasets (Figures 1d,e and S1f,g), indicating a strong link between AARS1 and aggressive disease biology as well as recurrence risk. At the cellular level, AARS1 expression was broadly higher in multiple bladder cancer cell lines than in normal urothelial cells, and immunofluorescence (IF) demonstrated strong AARS1 signals in tumor cells (Figure 1f,g).

We generated stable AARS1 overexpression and knockdown lines in T24 and UMUC3 cells (Figures 1h and S2a–d). Of note, the increase in AARS1 expression after lentiviral overexpression was moderate, likely because endogenous AARS1 expression was already relatively high in bladder cancer cells. We therefore selected stable overexpression populations within a biologically interpretable range rather than enforcing extreme supraphysiologic expression. Despite this moderate expression increase, AARS1 overexpression consistently enhanced malignant phenotypes, signaling output, metabolic rewiring, and in vivo tumor progression, supporting the functional relevance of this model. Functionally, AARS1 overexpression significantly enhanced colony formation, cell viability, and DNA synthesis, whereas AARS1 knockdown produced the opposite effects (Figures 1i,j and S2e–i). Cell‐cycle profiling further supported a pro‐proliferative role of AARS1 in driving cell‐cycle progression (Figure S2j). Moreover, AARS1 overexpression markedly promoted migration and invasion, while AARS1 silencing suppressed these phenotypes (Figures 1k and S3a–d). In parallel, AARS1 exerted an anti‐apoptotic effect. AARS1 overexpression reduced, whereas AARS1 knockdown increased, TUNEL positivity and Annexin V/7‐AAD–defined apoptosis (Figures 1l and S3e,f). Mechanistically, AARS1 induced an EMT shift, characterized by decreased E‐cadherin and increased N‐cadherin, Vimentin and SNAIL2. IF confirmed concordant spatial remodeling of E‐cadherin and N‐cadherin signals (Figures 1m,n and S3g–i). In vivo, subcutaneous xenografts and tail‐vein metastasis assays consistently demonstrated the tumor‐promoting role of AARS1. AARS1 overexpression increased tumor burden and lung colonization, while AARS1 depletion substantially suppressed tumor growth and metastatic load (Figures 1o–q and S3k–o). IHC of xenografts showed increased Ki67 and EMT features in AARS1‐high tumors (Figures 1p and S3j). To test whether AARS1‐driven oncogenic phenotypes depend on key functional residues, we performed rescue experiments in AARS1‐silenced cells by re‐expressing either AARS1‐WT or the catalytically impaired AARS1‐5A mutant (M46A/R77A/N216A/D239A/G241A). AARS1‐WT robustly restored proliferation, migration, invasion, apoptosis resistance and EMT remodeling, whereas AARS1‐5A showed markedly attenuated rescue capacity (Figure 1r–t; Figures S4a–m and S5a–f). Together, these data identify AARS1 as a clinically relevant driver of invasive and recurrent bladder cancer, with its pro‐tumor activities requiring intact functional residues represented by the 5A mutant.

### 3.2 AARS1 Is Required for Robust PI3K/AKT/mTOR Pathway Output in Bladder Cancer Cells

**FIGURE 2:**
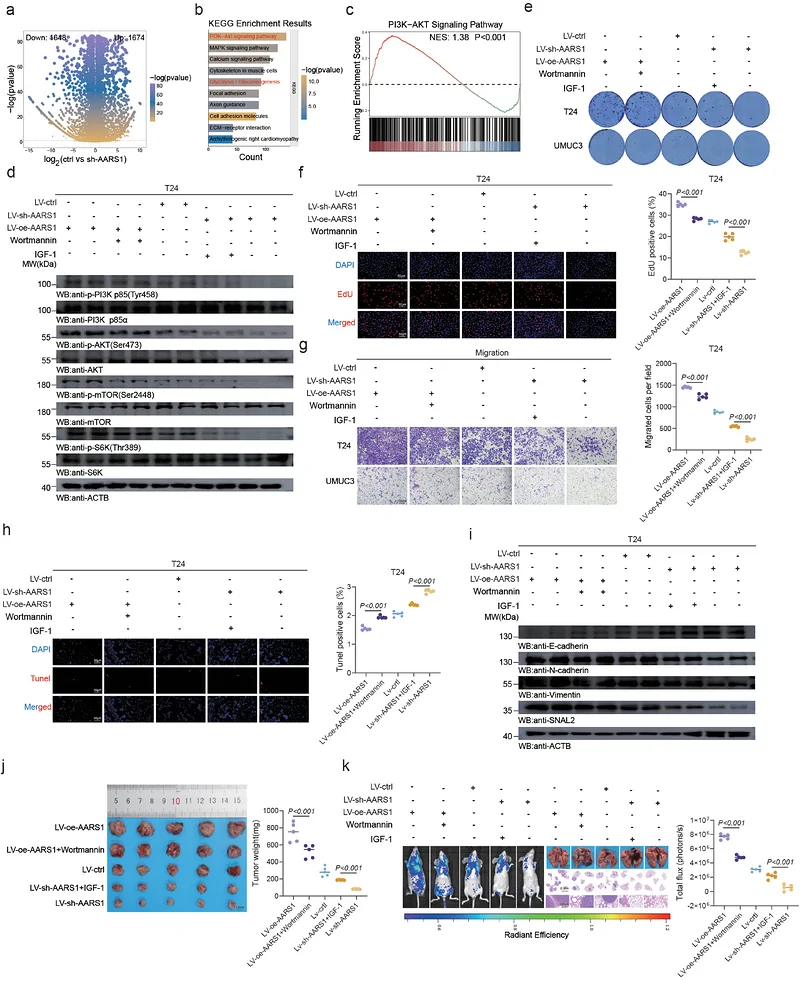
AARS1 activates the PI3K–AKT–mTOR cascade to drive malignant phenotypes in bladder cancer cells. (a) Volcano plot showing differentially expressed genes (DEGs) in AARS1‐silenced versus control T24 cells (RNA‐seq). (b) KEGG pathway enrichment analysis of DEGs, highlighting PI3K–AKT signaling among top‐enriched pathways. (c) Gene set enrichment analysis (GSEA) indicating significant association between AARS1 status and the PI3K–AKT signaling pathway (NES and *p* value as shown). (d) Immunoblot analysis of PI3K–AKT–mTOR pathway activation in T24 cells with AARS1 overexpression or knockdown, with pharmacological epistasis using the PI3K inhibitor wortmannin or IGF‐1 stimulation as indicated. (e) Representative colony formation assays of T24 and UMUC3 cells under the indicated genetic/pharmacological conditions. (f) EdU incorporation assay in T24 cells with representative images and quantification of EdU‐positive cells under the indicated conditions. (g) Transwell migration assays in T24 and UMUC3 cells with representative images and quantification of migrated cells per field. (h) TUNEL staining in T24 cells with representative images and quantification of TUNEL‐positive cells. (i) Immunoblot analysis of EMT‐associated proteins (E‐cadherin, N‐cadherin, vimentin, and SNAIL2) in T24 cells under the indicated conditions. (j) Subcutaneous xenograft model showing representative tumors and endpoint tumor weights from the indicated groups. (k) Tail‐vein metastasis model using luciferase‐labeled cells showing representative bioluminescence images, representative lungs and histology, and quantification of total photon flux. Scale bars are shown in the images. Data are presented as mean ± s.d.; statistical significance was determined using two‐tailed Student's *t* test (two‐group comparisons) or one‐way ANOVA with multiple‐comparisons correction (multi‐group comparisons), as appropriate. *P* values are indicated in the panels.

To define downstream programs governed by AARS1, we performed RNA‐seq in control and AARS1‐knockdown T24 cells. Differential expression analysis revealed broad transcriptome remodeling upon AARS1 depletion (Figure 2a). Both KEGG enrichment and GSEA consistently highlighted significant association with PI3K–AKT signaling (Figure 2b,c), nominating this axis as a central signaling hub downstream of AARS1. Immunoblotting confirmed that AARS1 overexpression enhanced phosphorylation of the PI3K–AKT–mTOR cascade (p‐PI3K, p‐AKT, p‐mTOR and p‐S6K), whereas AARS1 knockdown suppressed pathway activation (Figure S6a–d). Pharmacologic epistasis further placed AARS1 upstream of PI3K signaling. PI3K inhibitor Wortmannin abrogated AARS1 overexpression–induced pathway activation, while IGF‐1 stimulation partially restored signaling output in AARS1‐silenced cells (Figures 2d and S6e,f). To further clarify the signaling level at which AARS1 influences PI3K/AKT pathway activation, we examined receptor proximal signaling components in T24 and UMUC3 cells. AARS1 overexpression increased phosphorylation of EGFR, IGF1Rβ, IRS1, and AKT, whereas AARS1 knockdown reduced these signaling outputs. Wortmannin suppressed downstream AKT activation, while IGF‐1 stimulation partially restored receptor proximal signaling and AKT phosphorylation in AARS1 depleted cells (Figure S7). These findings support a functional association between AARS1 and receptor proximal PI3K/AKT pathway output. However, they do not establish direct biochemical regulation of the PI3K complex by AARS1. Consistently, Wortmannin significantly dampened AARS1 overexpression–driven colony formation and DNA synthesis, whereas IGF‐1 partially rescued the growth suppression induced by AARS1 knockdown (Figures 2e,f and S8a–e) and reversed corresponding cell‐cycle alterations (Figure S8f–h). Similarly, Wortmannin inhibited AARS1‐mediated migration and invasion, and IGF‐1 partially restored migratory and invasive capacities in AARS1‐depleted cells (Figures 2g and S9a–d). AARS1‐dependent apoptosis resistance was also PI3K sensitive. PI3K inhibition relieved the anti‐apoptotic effect of AARS1, while IGF‐1 partly reduced apoptosis triggered by AARS1 knockdown (Figures 2h and S9e–g). EMT marker remodeling followed the same reversible PI3K‐dependent pattern (Figures 2i and S10a–d). Importantly, these dependencies were validated in vivo. PI3K inhibition attenuated, and IGF‐1 partially restored, AARS1‐driven tumor growth and metastasis‐related phenotypes (Figures 2j,k and S10e–i). Collectively, these results indicate that AARS1 is required for robust PI3K/AKT/mTOR pathway output and is functionally associated with receptor proximal PI3K/AKT signaling in bladder cancer cells. This signaling relationship contributes to AARS1 associated proliferative, survival, and invasive phenotypes, while the direct receptor proximal or PI3K complex level mechanism remains to be further defined.

### 3.3 AARS1 Promotes PI3K‐Dependent Glycolytic Reprogramming and Lactate Production to Support Aggressive Behavior

**FIGURE 3:**
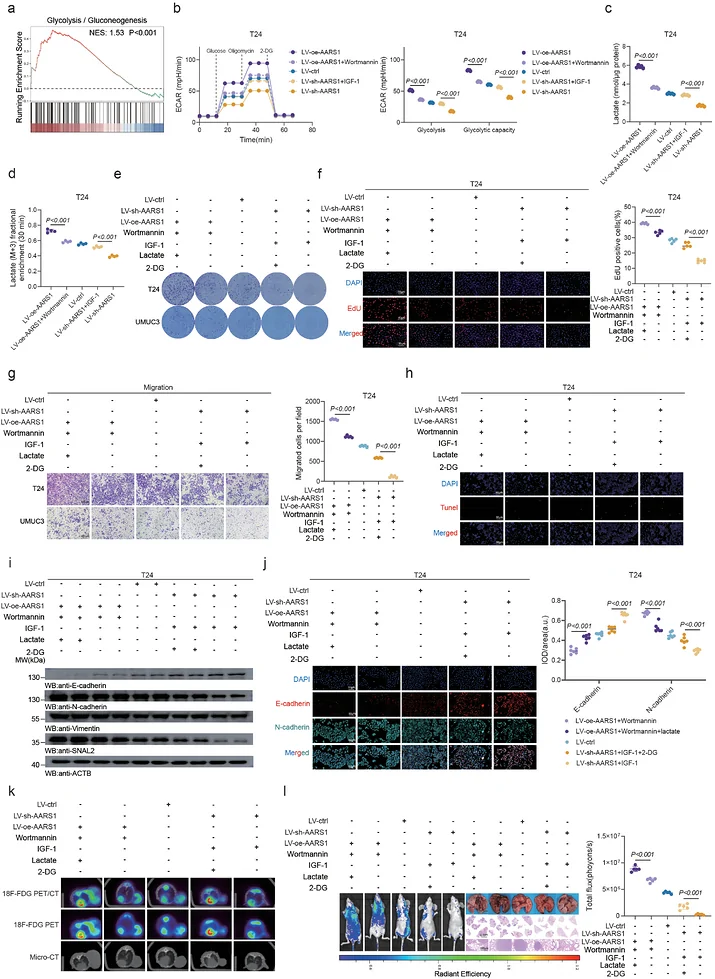
AARS1 drives PI3K‐dependent glycolytic reprogramming and lactate production to support malignant phenotypes. (a) Gene set enrichment analysis (GSEA) showing significant enrichment of the Glycolysis/Gluconeogenesis gene set in AARS1‐associated transcriptional programs (NES and *p* value as indicated). (b) Seahorse glycolysis stress test (ECAR) in T24 cells with AARS1 overexpression or knockdown, combined with wortmannin treatment or IGF‐1 stimulation as indicated. Left, representative ECAR time‐course following sequential injection of glucose, oligomycin, and 2‐deoxy‐D‐glucose (2‐DG); right, quantification of glycolysis and glycolytic capacity. (c) Lactate levels in culture supernatants (or cell lysates, as indicated) measured under the indicated conditions in T24 cells. (d) Uniformly labeled U‐^1^ ^3^C_6_‐glucose tracing showing fractional enrichment of lactate M+3 in T24 cells under the indicated conditions. (e) Colony formation assays in T24 and UMUC3 cells under the indicated genetic/pharmacological conditions, including metabolic epistasis with exogenous lactate supplementation or 2‐DG blockade. (f) EdU incorporation assay in T24 cells showing representative images and quantification of EdU‐positive cells under the indicated conditions. (g) Transwell migration assays in T24 and UMUC3 cells with representative images and quantification of migrated cells per field under the indicated conditions. (h) TUNEL staining in T24 cells showing representative images under the indicated conditions. (i) Immunoblot analysis of EMT‐associated proteins (E‐cadherin, N‐cadherin, vimentin, and SNAIL2) in T24 cells under the indicated conditions. (j) Immunofluorescence staining for E‐cadherin and N‐cadherin in T24 cells with representative images and quantification of fluorescence intensity (IOD/area) under the indicated conditions. (k) Representative ^1^ ^8^F‐FDG PET/CT images showing tumor glucose uptake under the indicated conditions. (l) Tail‐vein metastasis model using luciferase‐labeled cells showing representative bioluminescence images, representative lungs and histology, and quantification of total photon flux under the indicated conditions. Scale bars are shown in the images. Data are presented as mean ± s.d.; statistical significance was determined using two‐tailed Student's *t* test (two‐group comparisons) or one‐way ANOVA with multiple‐comparisons correction (multi‐group comparisons), as appropriate. *P* values are indicated in the panels.

Transcriptome enrichment suggested glycolysis‐related pathways as prominent AARS1‐associated programs (Figure 3a). We therefore assessed metabolic flux. Seahorse glycolysis stress tests showed that AARS1 overexpression significantly increased ECAR kinetics and elevated glycolysis and glycolytic capacity in T24 cells and these effects were attenuated by Wortmannin. Conversely, AARS1 knockdown reduced ECAR, which was partially restored by IGF‐1 (Figure 3b). Consistent results were obtained in UMUC3 cells (Figure S11a,b). Notably, OCR‐based mitochondrial respiration parameters remained largely unchanged across conditions (Figure S11k,l), suggesting that AARS1 preferentially enhances glycolytic flux rather than globally boosting oxidative phosphorylation. Consistent with increased glycolysis, lactate levels in conditioned media were significantly elevated upon AARS1 overexpression and decreased by Wortmannin. AARS1 knockdown reduced lactate, which was partially rescued by IGF‐1 (Figures 3c and S11c). U‐^1^ ^3^C_6_‐glucose tracing provided direct evidence of carbon rerouting to lactate. AARS1 increased lactate M+3 labeling, whereas PI3K inhibition or AARS1 depletion reduced the labeled fraction (Figures 3d and S11d). In contrast, isotopic labeling of multiple TCA cycle–related metabolites showed minimal changes (Figure S11e–j), further supporting a predominant shift toward the glycolysis to lactate axis.

Functionally, metabolic interventions reinforced causality. Exogenous lactate partially compensated the inhibitory effects of Wortmannin on colony formation and proliferation in AARS1‐overexpressing cells, whereas the ability of IGF‐1 to rescue growth defects in AARS1‐silenced cells was curtailed by glycolysis blockade with 2‐DG (Figures 3e,f and S12a–f). Migration, invasion and apoptosis phenotypes followed the same metabolic dependency (Figures 3g,h and S13a–i). EMT remodeling was tightly coupled to the PI3K–glycolysis–lactate axis. Wortmannin suppressed EMT features, lactate partially restored them, and IGF‐1–mediated rescue was constrained by 2‐DG (Figures 3i,j and S14a–c). In vivo, ^1^ ^8^F‐FDG PET/CT demonstrated significant changes in tumor glucose uptake consistent with the AARS1–PI3K–glycolysis program (Figures 3k and S14i), and these metabolic outputs aligned with changes in metastatic burden (Figures 3l and S14d–k). Together, AARS1 drives PI3K‐dependent glycolytic addiction and lactate accumulation, providing a metabolic foundation for tumor growth and dissemination.

### 3.4 Lactate‐Driven Lactylation Reprogramming Links AARS1 to H3K27la‐Dependent HK2 Activation and a Feed‐Forward Amplification Circuit

**FIGURE 4:**
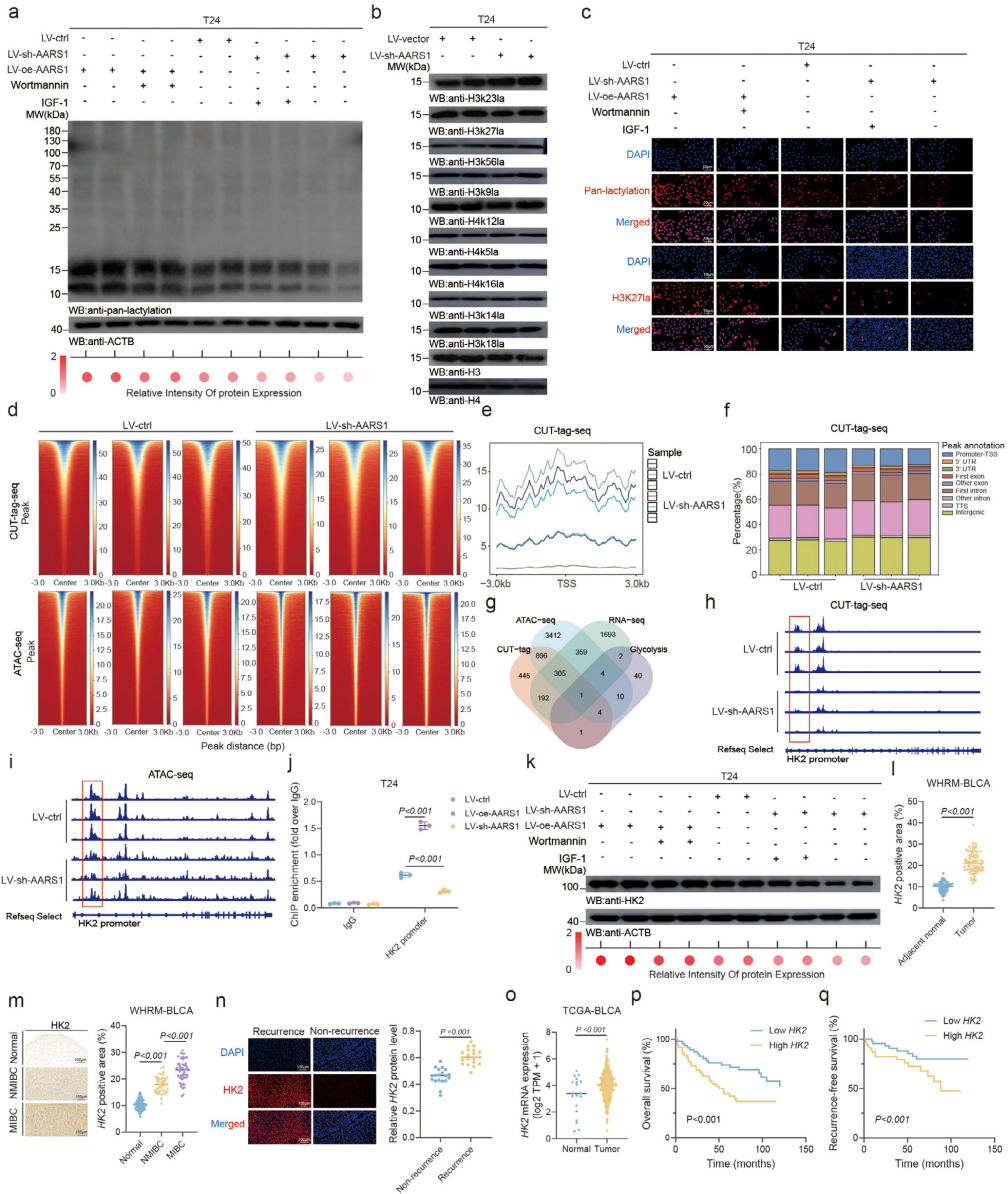
AARS1‐induced lactate accumulation rewires global and histone lactylation and activates HK2 transcription through H3K27la. (a) Immunoblotting of pan‐lysine lactylation (pan‐lactylation) in T24 cells with AARS1 overexpression or knockdown, combined with PI3K inhibition (wortmannin) or IGF‐1 stimulation as indicated. ACTB serves as a loading control. Densitometric summary is shown below. (b) Immunoblotting of multiple histone lactylation marks in T24 cells upon AARS1 knockdown, including H3K23la, H3K27la, H3K56la, H3K9la, H4K12la, H4K5la, H4K16la, H3K14la, and H3K18la. Histone H3 and H4 serve as loading controls. (c) Representative immunofluorescence images of nuclear pan‐lactylation and H3K27la in T24 cells under the indicated genetic and pharmacological conditions. Nuclei were counterstained with DAPI. (d) CUT‐Tag‐seq (H3K27la) and ATAC‐seq signal heatmaps aligned to transcription start sites (TSSs) in control versus AARS1‐silenced T24 cells. (e,g) Top, metagene profiles of H3K27la CUT‐Tag‐seq signal around TSSs in control and AARS1 knockdown cells. Bottom, Venn diagram showing overlap of candidate genes identified by differential H3K27la CUT‐Tag‐seq peaks, differential chromatin accessibility (ATAC‐seq), and RNA‐seq, highlighting enrichment of glycolysis‐related genes. (f) Genomic annotation of H3K27la CUT‐Tag‐seq peaks in control and AARS1 knockdown cells. (h) Genome browser tracks showing H3K27la CUT‐Tag‐seq signal at the HK2 promoter region in control and AARS1 knockdown cells. (i) Genome browser tracks showing ATAC‐seq signal at the HK2 promoter region in control and AARS1 knockdown cells. (j) ChIP–qPCR validation of H3K27la enrichment at the HK2 promoter in T24 cells, presented as enrichment over IgG. (k) Immunoblotting of HK2 protein levels in T24 cells with AARS1 overexpression or knockdown, with wortmannin or IGF‐1 as indicated. ACTB serves as a loading control; densitometric summary is shown below. (l) Quantification of HK2 immunohistochemical staining in the WHRM‐BLCA tissue microarray comparing adjacent normal and tumor tissues. (m) Representative IHC images and quantification of HK2 expression across normal, NMIBC, and MIBC samples in the WHRM‐BLCA cohort. (n) Representative immunofluorescence staining for HK2 in recurrence versus non‐recurrence tumors in the WHRM‐BLCA cohort, with corresponding quantification. (o) HK2 mRNA expression in TCGA‐BLCA tumor versus normal tissues. (p,q) Kaplan–Meier analyses of overall survival (p) and recurrence‐free survival (q) stratified by HK2 expression in the indicated cohort. Scale bars are shown in the images. Data are presented as mean ± s.d.; statistical significance was determined using two‐tailed Student's *t* test (two‐group comparisons) or one‐way ANOVA with multiple‐comparisons correction (multi‐group comparisons), as appropriate. *P* values are indicated in the panels.

Given lactate as a substrate for lactylation, we next tested whether AARS1‐induced lactate accumulation drives lactylation remodeling. Immunoblotting showed that AARS1 overexpression markedly increased pan‐lactylation, whereas AARS1 knockdown reduced global lactylation. Wortmannin diminished, and IGF‐1 partially restored, AARS1‐associated lactylation changes (Figures 4a and S15a–e), indicating tight coupling between lactylation and the AARS1–PI3K–glycolysis axis. To determine which histone lactylation marks were most responsive to AARS1 perturbation, we screened a panel of histone lactylation marks, including H3K23la, H3K27la, H3K56la, H3K9la, H3K14la, H3K18la, H4K5la, H4K12la, and H4K16la, with total H3 and H4 serving as loading controls. H3K18la was included in this screening panel. Among these marks, H3K27la showed the most consistent AARS1‐associated change across AARS1 depletion and overexpression models and was therefore selected for subsequent CUT‐Tag, ChIP‐qPCR, and HK2‐centered mechanistic validation (Figures 4b and S15f–i). IF confirmed reversible nuclear pan‐lactylation and H3K27la signals consistent with metabolic modulation (Figures 4c and S16a–j), supporting an epigenetic route by which lactate may be written onto chromatin to shape transcription. To identify downstream transcriptional outputs of H3K27la, we integrated H3K27la CUT‐Tag‐seq, ATAC‐seq and RNA‐seq. AARS1 depletion globally reduced H3K27la enrichment around transcription start sites (TSSs) (Figure 4d,e), and peak annotation showed stable localization across functional genomic regions including promoters (Figure 4f). Multi‐omics intersection prioritized glycolysis‐related candidates (Figure 4g). Among these, the HK2 promoter harbored a prominent H3K27la peak that was substantially diminished upon AARS1 knockdown (Figure 4h), and ATAC‐seq tracks supported concordant changes in chromatin accessibility at this locus (Figure 4i). ChIP–qPCR further validated H3K27la occupancy at the HK2 promoter, which increased with AARS1 upregulation and decreased with AARS1 depletion (Figures 4j and S17a–e). At the expression level, Wortmannin suppressed HK2 induction, whereas IGF‐1 or exogenous lactate partially restored HK2 under selected conditions, and 2‐DG constrained this output (Figures 4k and S17f–l). These findings support a feed‐forward metabolism–epigenetics–metabolism amplifier. Clinically, In malignancies, HK2 was noticeably higher than in surrounding tissues, increased along NMIBC‐to‐MIBC progression and in recurrent disease, and correlated with inferior OS and RFS (Figures 4l–q and S18a–d), consistent with this mechanistic model.

**FIGURE 5:**
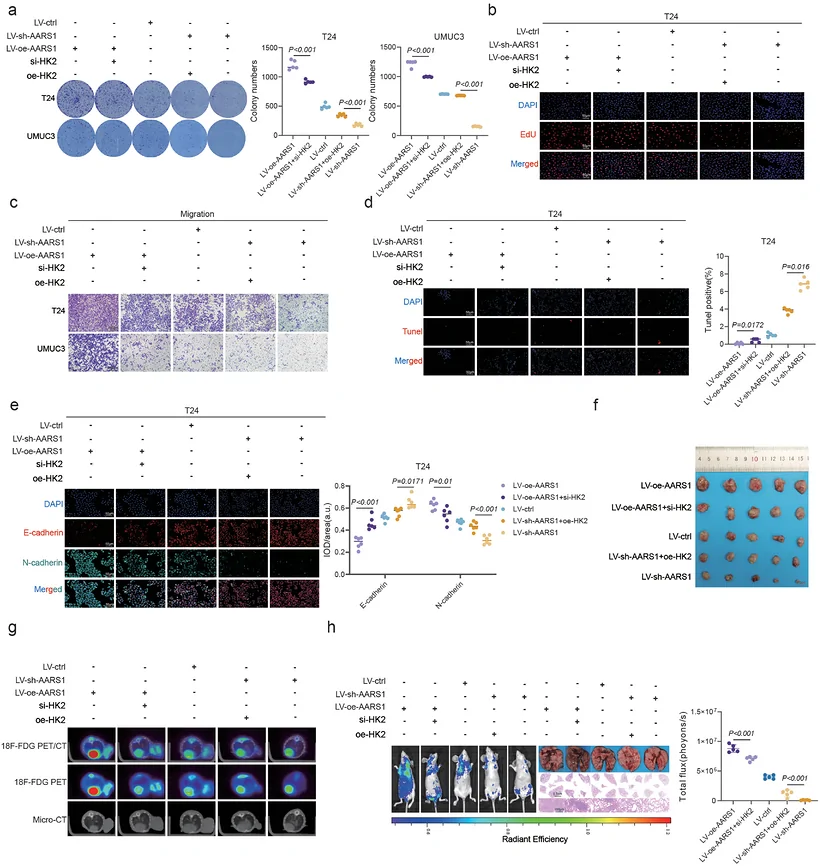
HK2 is a functional effector of AARS1 that mediates malignant phenotypes and metabolic outputs in bladder cancer cells. (a) Representative colony‐formation images and quantification in T24 and UMUC3 cells with genetic epistasis as indicated (AARS1 overexpression or knockdown combined with HK2 knockdown or overexpression). (b) Representative EdU incorporation images in T24 cells under the indicated genetic combinations. Nuclei were counterstained with DAPI. (c) Representative Transwell migration images in T24 and UMUC3 cells with AARS1/HK2 epistasis as indicated. (d) Representative TUNEL staining images and quantification of TUNEL‐positive cells in T24 cells under the indicated conditions. Nuclei were counterstained with DAPI. (e) Representative immunofluorescence staining of E‐cadherin and N‐cadherin in T24 cells with the indicated genetic combinations, with corresponding quantification of fluorescence intensity (IOD/area). Nuclei were counterstained with DAPI. (f) Representative images of subcutaneous xenograft tumors derived from T24 cells under the indicated genetic conditions (AARS1 gain‐ or loss‐of‐function with HK2 perturbation). (g) Representative ^1^ ^8^F‐FDG‐PET/CT and micro‐CT images of subcutaneous tumors from the indicated groups, showing differences in glucose uptake. (h) Representative bioluminescence images of mice and ex vivo lung images/representative H&E sections from experimental lung metastasis assays using luciferase‐labeled cells with AARS1/HK2 epistasis. Quantification of total flux (photons/s) is shown on the right. Data are presented as mean ± s.d. Statistical significance was determined using two‐tailed Student's *t* test (two‐group comparisons) or one‐way ANOVA with appropriate multiple‐comparisons correction (multi‐group comparisons), as applicable. Exact *p* values are indicated in the panels. Scale bars are shown in the images.

We next established the functional relevance of HK2 in vitro and in vivo. HK2 perturbation produced directionally consistent changes in proliferation, cell‐cycle progression, migration, invasion, apoptosis, and EMT markers, and these effects were validated in xenograft and lung colonization models (Figures S19–S21). Genetic epistasis further demonstrated that HK2 is a key effector of AARS1. HK2 knockdown in AARS1‐overexpressing cells significantly attenuated AARS1‐driven colony formation and proliferation (Figures 5a,b and S23a–g), migration and invasion (Figures 5c and S23h–j), apoptosis resistance (Figures 5d and S23k–m), and EMT remodeling (Figures 5e and S23n). To further test whether HK2 contributes to sustained lactate production and lactylation output downstream of AARS1, we performed HK2 rescue and depletion experiments in AARS1‐perturbed bladder cancer cells. HK2 re‐expression in AARS1‐depleted T24 and UMUC3 cells partially restored extracellular lactate levels, whereas HK2 silencing in AARS1‐overexpressing cells attenuated AARS1‐induced lactate production (Figure S22a,b). Consistently, HK2 re‐expression partially restored global protein lactylation and H3K27la in AARS1‐depleted cells, while HK2 silencing reduced pan‐lactylation and H3K27la in AARS1‐overexpressing cells (Figure S22c–f). These findings indicate that HK2 contributes to the maintenance of AARS1‐associated glycolytic and lactylation outputs, supporting an HK2‐centered metabolic and epigenetic reinforcement model. In vivo, HK2 similarly dictated metabolic and metastatic outputs. ^1^ ^8^F‐FDG PET/CT and bioluminescence imaging showed that HK2 depletion mitigated, whereas HK2 re‐expression partially restored, AARS1‐dependent glucose uptake and metastatic progression (Figures 5f–h and S24a–g). Collectively, these data identify HK2 as a critical transcriptional effector of the AARS1–lactate–H3K27la axis and demonstrate that H3K27la‐mediated HK2 activation sustains a self‐reinforcing glycolytic addiction program.

### 3.5 Drug Repurposing Identifies Eltrombopag as an AARS1‐Binding Compound That Suppresses the AARS1‐Associated Metabolic and Epigenetic Program and Restrains Tumor Progression

**FIGURE 6:**
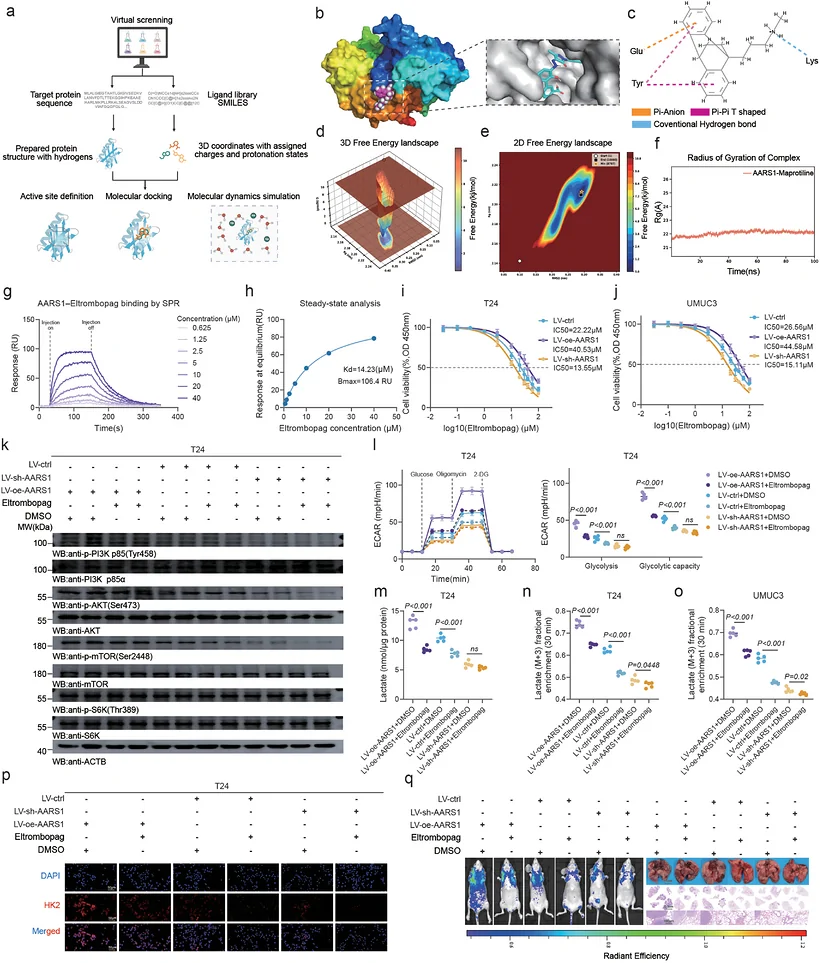
Drug repurposing identifies Eltrombopag as an AARS1‐binding compound that suppresses the AARS1‐associated metabolic and epigenetic program and restrains tumor progression. (a) Schematic workflow of structure‐guided drug repurposing, including protein preparation, binding‐site definition, virtual screening/molecular docking, and molecular dynamics (MD) simulation. (b) Predicted binding pose of Eltrombopag within the AARS1 pocket shown in surface representation, with a zoomed‐in view of the ligand accommodated in the cavity. (c) Two‐dimensional interaction map summarizing key noncovalent contacts between Eltrombopag and AARS1 (including hydrogen bonds and π interactions, as indicated). (d,e) Representative 3D and 2D free‐energy landscapes derived from MD simulation, supporting a stable bound‐state basin for the AARS1–Eltrombopag complex. (f) Radius of gyration (Rg) of the AARS1–Eltrombopag complex over the MD trajectory, indicating overall conformational stability during simulation. (g,h) Surface plasmon resonance (SPR) validation of direct binding between Eltrombopag and AARS1. Representative sensorgrams acquired across an Eltrombopag concentration series (0.625–40 µM) and steady‐state fitting to estimate binding affinity (KD shown in panel). (i,j) Dose–response curves of Eltrombopag in T24 (i) and UMUC3 (j) cells with control, AARS1 overexpression, or AARS1 knockdown backgrounds; IC50 values are indicated in the panels. (k) Immunoblot analysis showing that Eltrombopag suppresses AARS1‐associated activation of the PI3K–AKT–mTOR pathway (p‐PI3K, p‐AKT, p‐mTOR, and p‐S6K), with total proteins and ACTB as loading controls, under the indicated genetic and treatment conditions (DMSO as vehicle). (l) Seahorse glycolysis stress test (ECAR) in T24 cells following glucose, oligomycin, and 2‐DG injection, with quantification of glycolysis and glycolytic capacity under the indicated conditions (DMSO vs Eltrombopag; AARS1 gain‐ or loss‐of‐function backgrounds). (m) Lactate levels normalized to protein content in T24 cells under the indicated conditions. (n,o) U‐^1^ ^3^C_6_‐glucose tracing showing the fractional enrichment of lactate M+3 in T24 (n) and UMUC3 (o) cells, indicating that Eltrombopag reduces glucose‐to‐lactate carbon flux. (p) Representative immunofluorescence staining of HK2 in T24 cells under the indicated genetic and treatment conditions; nuclei were counterstained with DAPI. (q) In vivo experimental lung metastasis assays using luciferase‐labeled cells with the indicated AARS1 perturbations and treatments (Eltrombopag vs DMSO), showing representative bioluminescence images, ex vivo lung images, representative H&E sections, and quantification of total flux (photons/s). Data presentation and statistical analyses follow the Methods; exact *p* values (or significance as indicated) are shown in the panels. Scale bars are shown in the images.

Based on the above mechanistic findings, we asked whether pharmacologic perturbation of the AARS1‐associated program could suppress PI3K signaling, glycolysis, lactate output, and downstream lactylation‐associated transcriptional events. Structure‐guided virtual screening, molecular docking, and molecular dynamics simulations predicted that Eltrombopag stably occupied a potential AARS1‐binding pocket through multiple noncovalent interactions, with a stable conformational profile during simulation(Figure 6a–f). Surface plasmon resonance provided direct biophysical evidence that Eltrombopag binds recombinant AARS1 with micromolar affinity(Figure 6g,h). Dose–response analyses further demonstrated robust growth‐inhibitory activity in T24 and UMUC3 cells, with responses consistent with AARS1 dependency (Figure 6i,j). Mechanistically, Eltrombopag markedly reduced activation of the PI3K–AKT–mTOR cascade, as reflected by decreased p‐PI3K, p‐AKT, p‐mTOR and p‐S6K with relatively modest changes in total protein levels (Figures 6k and S25a–c). Metabolically, Eltrombopag significantly suppressed ECAR and glycolytic capacity (Figures 6l and S26a–f) and decreased lactate abundance (Figures 6m and S26g). U‐^1^ ^3^C_6_‐glucose tracing demonstrated that Eltrombopag preferentially reduced glucose‐derived carbon flux into lactate (lactate M+3), while isotopologue patterns of several TCA‐related metabolites were minimally affected (Figures 6n,o and S26h–m), consistent with selective disruption of the glycolysis to lactate axis. Critically, Eltrombopag also blocked the epigenetic output of the circuit. In both T24 and UMUC3 cells, Eltrombopag reduced global pan‐lactylation and H3K27la signals and suppressed HK2 protein expression (Figure 6p; Figures S27a–f and S28a–e). Accordingly, Eltrombopag inhibited proliferation and clonogenicity, reduced EdU incorporation and altered cell‐cycle distribution (Figure S29a–g), suppressed migration and invasion (Figure S30a–f), and increased apoptosis (Figure S30g–k). EMT‐associated proteins shifted toward an epithelial phenotype at both immunoblot and IF levels (Figure S31a–d), supporting the anti‐metastatic mechanism.

In vivo, Eltrombopag significantly reduced lung metastatic burden, as evidenced by decreased bioluminescence total flux, fewer gross lung nodules, and diminished metastatic lesions on histology (Figures 6q and S32i–k). Subcutaneous xenograft experiments further showed that eltrombopag suppressed tumor growth without overt body‐weight loss, suggesting acceptable tolerability (Figure S32a–c). Tumor IHC confirmed reduced Ki67 and concomitant downregulation of pan‐lactylation, H3K27la and HK2, together with EMT marker reversion (Figure S32d–h). Collectively, these results identify Eltrombopag as an AARS1‐binding compound that pharmacologically suppresses the AARS1‐associated PI3K–glycolysis–lactate–lactylation–HK2 program and restrains bladder cancer progression in vitro and in vivo. Although these findings support Eltrombopag as a candidate AARS1‐targeting agent for further investigation, direct inhibition of AARS1 enzymatic activity and cellular target specificity remain to be established.

## 4 Discussion

BC remains clinically challenging due to high recurrence rates in NMIBC, frequent progression to invasive disease, and limited durability of systemic therapies in advanced settings [4, 21]. Here we identify AARS1 as a clinically and functionally relevant node associated with oncogenic signaling, metabolic rewiring, and epigenetic reinforcement in aggressive and recurrence‐prone BC states. Across patient cohorts, AARS1 is consistently upregulated, enriched in invasive and recurrent tumors, and associated with unfavorable survival, supporting its clinical relevance beyond a mere metabolic correlate. Functionally, AARS1 promotes proliferation, EMT, survival and metastatic colonization, and these oncogenic outputs require intact AARS1 functional residues, supporting further therapeutic exploration of AARS1‐associated vulnerabilities. A key conceptual advance of this work is the identification of an AARS1‐associated metabolic and epigenetic reinforcement program that links PI3K pathway output, glycolytic lactate production, and chromatin regulation in aggressive bladder cancer. Our data show that AARS1 is required for robust PI3K–AKT–mTOR pathway activation in this context and is associated with enhanced glycolytic flux and lactate accumulation, while oxidative respiration is less prominently affected. PI3K inhibition blunts AARS1‐associated signaling and phenotypes, whereas IGF‐1 partially restores outputs under AARS1 loss, supporting a functional relationship between AARS1 and PI3K‐dependent malignant programs. These findings nominate AARS1 as a potential regulatory node connecting oncogenic signaling to metabolic commitment.

Beyond lactate as a by‐product, our data place lactate at the center of a metabolism‐to‐chromatin writing mechanism that stabilizes malignant transcriptional states. AARS1‐induced lactate elevation is accompanied by increased global protein lactylation and, notably, histone lactylation, with H3K27la emerging as a dominant responsive mark. Multi‐omics integration converges on HK2 as a prominent downstream candidate linked to H3K27la‐associated chromatin remodeling. AARS1 depletion reduces H3K27la enrichment around TSSs and diminishes H3K27la occupancy and chromatin accessibility at the HK2 promoter, coupled with decreased HK2 expression. Orthogonal ChIP–qPCR validation supports promoter‐level enrichment of H3K27la at this locus. Genetic epistasis further demonstrates that HK2 is a functionally important effector of AARS1‐associated proliferation, invasion, apoptosis resistance, EMT, glucose uptake, and metastatic burden. Collectively, these findings support a feed‐forward model in which AARS1‐associated PI3K pathway output enhances glycolytic flux and lactate accumulation, thereby increasing H3K27 lactylation at the HK2 promoter and supporting HK2 transcription. Elevated HK2 may then reinforce glycolysis and lactate production, forming an HK2‐centered metabolic and epigenetic reinforcement program that may help explain the persistence of aggressive, recurrence‐ and progression‐associated phenotypes. It is also important to note that H3K18la has been implicated in bladder cancer cisplatin resistance, suggesting that distinct histone lactylation marks may dominate under different biological or therapeutic contexts [22]. In our AARS1 perturbation and recurrence‐oriented models, H3K27la showed the most consistent AARS1‐associated change and was therefore prioritized for mechanistic analysis. Future studies using cisplatin‐treated or cisplatin‐resistant bladder cancer models will be needed to determine whether AARS1 also modulates H3K18la‐dependent transcriptional programs and whether H3K18la cooperates with or diverges from the H3K27la/HK2 axis during therapy adaptation. In addition to HK2, pyruvate dehydrogenase kinase isoforms PDK1 to PDK4 should also be considered as important regulators of cancer metabolic reprogramming and lactate accumulation in bladder cancer [23]. PDKs phosphorylate and inhibit the pyruvate dehydrogenase complex, thereby limiting pyruvate entry into mitochondrial oxidative metabolism and favoring pyruvate diversion toward lactate production. This mechanism is consistent with the broader Warburg phenotype observed in bladder cancer, and previous studies have shown that PDK4 is markedly upregulated in high grade bladder cancer and contributes to tumor growth and chemoresistance [24]. Therefore, PDK family members may represent parallel or cooperative regulators of the lactate producing metabolic state. In the present study, we prioritized HK2 because it emerged at the intersection of differential H3K27la occupancy, altered chromatin accessibility, and RNA‐seq based transcriptional regulation, and because HK2 perturbation functionally modified AARS1 associated glycolytic, lactylation, and malignant phenotypes. Thus, HK2 represents the most directly supported downstream effector of the AARS1‐associated H3K27la program in our dataset, while PDK family members may act as parallel or cooperative metabolic regulators that sustain lactate accumulation in bladder cancer. Although our mechanistic analysis focused on the H3K27la/HK2 chromatin axis, AARS1‐associated lactylation is likely to involve a broader substrate spectrum. Recent evidence indicates that AARS1 can function as a lactate‐responsive lactyltransferase and promote non‐histone lactylation of p53, suggesting that AARS1 may influence tumor behavior through both chromatin‐dependent and chromatin‐independent mechanisms [20, 25]. Although these pathways have not been directly tested in our bladder cancer models, p53‐related stress responses, STAT‐family inflammatory signaling, and YAP/TEAD‐driven transcriptional plasticity are biologically relevant to proliferation, immune modulation, invasion, and treatment resistance [26, 27]. These pathways therefore represent plausible non‐histone branches of the AARS1‐associated lactylation network. However, the present study did not directly map AARS1‐dependent non‐histone lactylation substrates. Future studies using quantitative lactylome profiling, immunoprecipitation‐based validation of candidate lactylation sites, site‐mutant rescue models, and pathway‐specific functional assays will be needed to determine whether p53, STAT1, YAP/TEAD, or other non‐histone targets cooperate with the H3K27la/HK2 program to sustain aggressive and recurrent bladder cancer states.

Our work also extends the expanding non‐canonical repertoire of aaRSs in cancer. While aaRSs are classically viewed as housekeeping enzymes for translation, emerging studies highlight broader roles in signaling and tumor biology, and recent evidence suggests AARS1 can couple lactate to lysine lactylation of cellular proteins [18, 28]. In bladder cancer, our data indicate that AARS1 coordinates both lactate supply and chromatin‐level lactylation output to stabilize a glycolysis‐dependent, invasive program. By promoting lactate production through PI3K‐dependent glycolysis while simultaneously enabling lactylation‐based transcriptional reinforcement, AARS1 provides a mechanistic explanation for the magnitude and persistence of the phenotypes observed. This dual control suggests that AARS1 upregulation can consolidate malignant cell states rather than merely supporting short‐lived metabolic adaptation. The regulatory framework governing lactylation is still developing, and our findings contribute disease‐relevant evidence to this emerging field. Delactylase activities reported for HDAC family members support the view that lactylation is dynamic and reversible, whereas accumulating biochemical observations suggest that lactylation control may be context dependent and may not follow a single linear pathway [29, 30, 31, 32]. Within this evolving landscape, our results identify H3K27 lactylation at the HK2 promoter as a sensitive and reversible regulatory node that links lactate availability to chromatin activity. The observation that PI3K inhibition and glycolysis blockade attenuate both H3K27 lactylation and HK2 expression argues against a passive association and instead supports a model in which this locus functions as a metabolically gated epigenetic mechanism. Future studies that define the enzymatic regulators responsible for H3K27 lactylation in bladder cancer, using genetic perturbation, pharmacologic epistasis, and locus‐resolved chromatin assays, will be important to transform the current mechanistic framework into a fully specified enzymatic circuit and to expand therapeutic opportunities. Although PI3K, AKT, and mTOR pathway alterations are prevalent in bladder cancer, clinical inhibition of this axis is often constrained by toxicity and adaptive resistance [33, 34, 35]. AARS1 may provide a distinct leverage point because targeting this node could attenuate PI3K pathway output, glycolytic flux, lactate generation, and lactylation‐dependent transcriptional reinforcement. Consistent with this rationale, we pursued a drug repurposing strategy and identified Eltrombopag as an AARS1‐binding compound supported by structure‐guided docking, molecular dynamics simulation, and direct binding measured by SPR. Functionally, Eltrombopag suppressed PI3K, AKT, and mTOR activation, reduced glucose‐to‐lactate carbon flux, decreased global lactylation and H3K27 lactylation, downregulated HK2, and attenuated malignant phenotypes and metastatic progression in vivo. These pharmacologic effects are concordant with the genetically defined AARS1‐associated program and support Eltrombopag as a candidate AARS1‐targeting agent for further investigation. However, the present data do not establish direct inhibition of AARS1 enzymatic activity or complete target specificity in cells. Future studies using cellular thermal shift assay, drug affinity responsive target stability assay, chemoproteomic profiling, in vitro AARS1 lactyltransferase activity assays, and resistance‐conferring AARS1 variants will be essential to define target engagement, target dependency, and the precise inhibitory mechanism required for translational development.

Several limitations should be acknowledged. First, although our data support a functional association between AARS1 and PI3K pathway output, including altered receptor proximal signaling involving EGFR, IGF1R, IRS, and AKT, the receptor proximal or PI3K complex level mechanism linking AARS1 to this pathway remains to be defined. Future studies examining receptor tyrosine kinase activation, PI3K complex assembly, PIP3 dynamics, and potential protein interactions will be needed to clarify this regulatory layer. Second, although Eltrombopag was identified as an AARS1‐binding compound and suppresses the AARS1‐associated metabolic and epigenetic program, the present data do not establish direct inhibition of AARS1 enzymatic activity or complete target specificity in cells. cellular thermal shift assay (CETSA), drug affinity responsive target stability assay (DARTS), chemoproteomic profiling, in vitro lactyltransferase assays, and resistance‐conferring AARS1 variants will be required to define target engagement and target dependency. Third, this study focused mainly on tumor cell‐intrinsic mechanisms, and immunodeficient xenograft models may underrepresent immune‐metabolic interactions driven by lactate and lactylation. Future studies in immunocompetent or humanized systems will be important to evaluate the immune relevance of AARS1‐associated metabolic reprogramming and potential combinations with immune checkpoint blockade. Fourth, prospective validation in multicenter cohorts with harmonized treatment histories will be necessary to determine the prognostic and therapeutic relevance of AARS1 and HK2 in bladder cancer. Finally, although Eltrombopag represents a practical starting point, oncology‐oriented optimization may be required, including selectivity assessment, structure‐guided refinement, pharmacokinetic and pharmacodynamic matching, and direct target‐engagement validation, to develop more selective AARS1‐targeting strategies for bladder cancer treatment.

## 5 Conclusions

In conclusion, we identify AARS1 as a clinically relevant driver of aggressive and recurrence associated bladder cancer. AARS1 promotes PI3K AKT mTOR pathway output, glycolytic reprogramming, and lactate accumulation, which are linked to increased H3K27 lactylation at the HK2 promoter and HK2 transcriptional activation. This AARS1 associated metabolic and epigenetic program supports glycolytic reinforcement and malignant phenotypes, including proliferation, EMT, apoptosis resistance, and metastatic colonization. Importantly, eltrombopag, identified by structure guided screening and SPR validation as an AARS1 binding compound, pharmacologically suppresses the AARS1 associated PI3K, glycolysis, lactate, lactylation, and HK2 program and restrains tumor growth and lung metastasis in vivo. These findings nominate AARS1 as a potential therapeutic vulnerability in bladder cancer and support eltrombopag as a practical starting point for future development of AARS1 targeting strategies, pending more definitive validation of target engagement, enzymatic inhibition, and target specificity.

## Author Contributions

**Qin Yuan** (QY), **Tianbao Song** (TS), and **Yipeng He** (YH) conceived and designed the study. QY, TS, and YH performed the majority of the experiments, analyzed the data, and drafted the manuscript. **Sihan Xia** (SX) conducted the bioinformatics analyses, including RNA‐seq, CUT&Tag‐seq/ATAC‐seq integration, and related statistical analyses. **Yaoqiu Huang** (YHu) and **Wenlin He** (WH) assisted with mechanistic experiments and supported the validation assays. **Qin Yi** (QYi), **Zefeng Wang** (ZW), **Peihan Wang** (PW), **Chenbo Huang** (CH), **Linzhi Li** (LL), and **Zhen Yin** (ZY) contributed to sample collection/processing, experimental execution, data acquisition, and technical support. **Fan Cheng** (FC) and **Weimin Yu** (WY) provided overall supervision, secured funding, and critically revised the manuscript. All authors reviewed and approved the final manuscript.

## Funding

This work was supported by grants from the National Natural Science Foundation of China (Nos. 82170775 and 82370765).

## Ethics Statement

Human studies were approved by the Institutional Review Board of Renmin Hospital of Wuhan University, with informed consent waived due to the retrospective design. All animal experiments were approved by the Institutional Animal Care and Use Committee of Renmin Hospital of Wuhan University.

## Conflicts of Interest

The authors declare no conflicts of interest.

## Supporting information




**Supporting File 1**: advs77479‐sup‐0001‐SuppMat.docx.

**Supporting File 2**: advs77479‐sup‐0002‐SuppMat.docx.

## Data Availability

The data that support the findings of this study are available from the corresponding author upon reasonable request.
