# Supplementary material for: AARS1‐Mediated H3K27 Lactylation Rewires Glycolysis to Sustain Aggressive and Recurrent Bladder Cancer
Source: Adv Sci (Weinh). 2026 Aug 30:e77479. Online ahead of print. doi: 10.1002/advs.77479 (PMC13526531; doi:10.1002/advs.77479)
Supplement: Supplementary file 1 [file ADVS-9999-e77479-s001.docx]

**Supplementary Table S1. The Detailed information of antibody used in the experiment**

| Antibody | Species | Application | Catalog Number | Company | Country | Dilution |
| --- | --- | --- | --- | --- | --- | --- |
| Beta Actin | Mouse | WB | 66009-1-Ig | Proteintech | China | 1:10000 |
| AARS1 | Rabbit | WB,  IF,  IHC | 17394-1-AP | Proteintech | China | 1:3000  1:200  1:200 |
| VIM | Rabbit | WB | 10366-1-AP | Proteintech | China | 1:2000 |
| SNAI2 | Rabbit | WB | 12129-1-AP | Proteintech | China | 1:1000 |
| N-cadherin | Rabbit | WB,  IF,  IHC | 22018-1-AP | Proteintech | China | 1:5000  1:200  1:2000 |
| E-cadherin | Rabbit | WB,  IF,  IHC | 20874-1-AP | Proteintech | China | 1:20000  1:200  1:5000 |
| HK2 | Rabbit | WB,  IF,  IHC | 22029-1-AP | Proteintech | China | 1:10000  1:200  1:1000 |
| p-PI3K | Rabbit | WB | AF3242 | Affinity Biosciences | China | 1:1000 |
| PI3K | Rabbit | WB | AF6241 | Affinity Biosciences | China | 1:1000 |
| p-AKT | Mouse | WB | 66444-1-AP | Proteintech | China | 1:2000 |
| AKT | Rabbit | WB | 10176-1-AP | Proteintech | China | 1:2000 |
| p-mTOR | Mouse | WB | 67778-1-AP | Proteintech | China | 1:2000 |
| mTOR | Mouse | WB | 66888-1-AP | Proteintech | China | 1:5000 |
| p-S6K | Rabbit | WB | 81592-1-AP | Proteintech | China | 1:5000 |
| S6K | Mouse | WB | 66638-1-AP | Proteintech | China | 1:1000 |
| H3 | Rabbit | WB | 17168-1-AP | Proteintech | China | 1:1000 |
| H4 | Rabbit | WB | 16047-1-AP | Proteintech | China | 1:1000 |
| DAPI | Rabbit | IF | 28718-90-3 | Proteintech | China | 1:1 |
| Ki-67 | Rabbit | IHC | 27309-1-AP | Proteintech | China | 1:4000 |
| Pan-KLA | Rabbit | WB,  IF,  IHC | PTM-1401RM | PTM-BIO | China | 1:1000  1:100  1:200 |
| H3K23a | Rabbit | WB | PTM-1413RM | PTM-BIO | China | 1:2000 |
| H3K9la | Rabbit | WB | PTM-1419RM | PTM-BIO | China | 1:1000 |
| H3K14la | Rabbit | WB | PTM-1414RM | PTM-BIO | China | 1:1000 |
| H4K12la | Rabbit | WB | PTM-1411RM | PTM-BIO | China | 1:1000 |
| H3K27la | Rabbit | WB,  IF,  IHC,  CUT-tag | PTM-1428RM | PTM-BIO | China | 1:2000  1:100  1:200  1:50 |
| H4K5la | Rabbit | WB | PTM-1414RM | PTM-BIO | China | 1:1000 |
| H3K56la | Rabbit | WB | PTM-1421RM | PTM-BIO | China | 1:1000 |
| H4K16la | Rabbit | WB | PTM-1417RM | PTM-BIO | China | 1:1000 |
| H3K18la | Rabbit | WB | PTM-1406RM | PTM-BIO | China | 1:1000 |
| p-IGF1Rβ | Rabbit | WB | 3024 | CST | America | 1:1000 |
| IGF1Rβ | Rabbit | WB | 9750 | CST | America | 1:1000 |
| IRS1 | Rabbit | WB | 17509-1-AP | Proteintech | China | 1:2000 |
| p-IRS1 | Rabbit | WB | 85238-1-AP | Proteintech | China | 1:2000 |
| EGFR | Rabbit | WB | 18986-1-AP | Proteintech | China | 1:1000 |
| p-EGFR | Rabbit | WB | 30278 | Proteintech | China | 1:1000 |
| IgG | Rabbit | CUT-tag | 30000-0-AP | Proteintech | China | 1:100 |
| Goat Anti-Rabbit IgG | Goat | WB | SA00001-2 | Proteintech | China | 1:10000 |
| Goat Anti-Mouse IgG(H+L) | Goat | WB | SA00001-1 | Proteintech | China | 1:10000 |

**Supplementary Table S2. siRNA sequences used in this study.**

| Gene | Sense (5′-3′) | Antisense (5′-3′) |
| --- | --- | --- |
| HK2 | CACGATGAAATTGAACCTGGT | ACCAGGTTCAATTTCATCGTG |

**Supplementary Table S3. shRNA sequences used in this study.**

| Gene | Target sequence (5'–3') | Vector |
| --- | --- | --- |
| AARS1 | GCAGCGATTTATAGATTTCTT | pLKO.1 |
| HK2 | GCTACAAATCAAAGACAAGAA | pLKO.1 |

**Supplementary Table S4. Key small molecules and recombinant protein used in this study.**

| Reagent | Purpose | Supplier and catalog number | Solvent or preparation | Stock concentration | Working concentration | Treatment duration or usage |
| --- | --- | --- | --- | --- | --- | --- |
| Wortmannin | PI3K inhibition | MedChemExpress; HY-10197 | DMSO | 10 mM | 0.5 μM | 24 h for signaling, metabolism, lactylation and functional rescue assays; In vivo: 0.6 mg/kg, intraperitoneal injection, twice weekly |
| Recombinant human IGF-1 | IGF-1 pathway activation | PeproTech; 100-11 | Sterile PBS containing 0.1% BSA | 100 μg/mL | 50 ng/mL | 24 h for rescue of AARS1 knockdown related phenotypes; In vivo: 1 mg/kg, tail-vein injection, once daily |
| 2-Deoxy-D-glucose | Glycolysis blockade | Sigma-Aldrich; D8375 | Sterile water | 1 M | 5 mM | 24 h for glycolysis inhibition and functional rescue assays |
| 2-Deoxy-D-glucose | Seahorse glycolysis stress test | Sigma-Aldrich; D8375 | Seahorse assay medium | Prepared according to Seahorse protocol | 50 mM final concentration | Acute injection during ECAR measurement |
| Sodium L-lactate | Lactate supplementation | Sigma-Aldrich; L7022 | Sterile water or PBS, pH adjusted to 7.4 | 1 M | 10 mM | 24 h for lactate rescue assays |
| Eltrombopag | Pharmacologic suppression of the AARS1 associated program | MedChemExpress; HY-15306 | DMSO for in vitro assays | 20 mM | 5, 10 and 20 μM | 24 to 48 h according to the assay; 24 h for signaling, metabolism and lactylation assays; 48 h for viability assays; In vivo: 75 mg/kg, intraperitoneal injection, every three days |
| Eltrombopag | SPR binding assay | MedChemExpress; HY-15306 | Running buffer containing matched DMSO | 20 mM | 0.625 to 40 μM | Injected for 60 s association and 180 s dissociation at 30 μL/min |
| DMSO | Vehicle control for small molecules | Sigma-Aldrich; D2650 | Ready to use | 100% | ≤0.1% v/v in culture medium | Same duration as corresponding drug treatment; In vivo: Used as vehicle when applicable |
| PBS | Vehicle control and cell suspension | Gibco; 10010023 | Ready to use | 1× | 1× | Equal volume vehicle control or cell suspension; In vivo: Used as vehicle when applicable |
| Puromycin | Selection of stable cell lines | Gibco; A1113803 | Sterile water | 10 mg/mL | 2 μg/mL for selection; 1 μg/mL for maintenance | 3 to 7 days for selection; maintained as needed |
| Polybrene | Lentiviral transduction enhancement | Sigma-Aldrich; H9268 | Sterile water | 10 mg/mL | 8 μg/mL | 12 to 24 h during lentiviral transduction |
| CCK-8 reagent | Cell viability assay | Dojindo; CK04 | Ready to use | Ready to use | 10 μL per well in 96-well plates | Incubation for 2 h before absorbance measurement at 450 nm |
| EdU reagent | DNA synthesis assay | Thermo Fisher Scientific; C10337 | Prepared according to kit protocol | According to kit protocol | 10 μM | 2 h pulse labeling |
| Crystal violet | Colony formation and Transwell staining | Sigma-Aldrich; C0775 | Water or 20% methanol | 0.5% to 1% stock | 0.1% | 15 to 30 min staining after fixation |
| Propidium iodide | Cell cycle analysis | Sigma-Aldrich; P4170 | PBS | 1 mg/mL | 50 μg/mL | 30 min staining after RNase A treatment |
| RNase A | Cell cycle analysis | Thermo Fisher Scientific; EN0531 | Water | 10 mg/mL | 100 μg/mL | 30 min at 37 °C before PI staining |
| Annexin V/7-AAD apoptosis kit | Apoptosis assay | BD Biosciences; 559763 | Provided in kit | Ready to use | According to kit protocol | 15 min at room temperature, protected from light |
| TUNEL kit | Apoptosis detection | Roche; 11684795910 | Provided in kit | Ready to use | According to kit protocol | According to manufacturer’s instructions |
| Matrigel | Transwell invasion assay | Corning; 356234 | Thawed on ice, diluted in serum-free medium | Ready to use | 1:8 dilution | Coating of Transwell inserts before cell seeding |
| U-¹³C₆ glucose | Isotope tracing | Cambridge Isotope Laboratories; CLM-1396 | Glucose-free culture medium | Prepared fresh | 10 mM | 6 h tracing in medium containing 10% dialyzed FBS |
| Dialyzed FBS | Isotope tracing | Gibco; A3382001 | Ready to use | Ready to use | 10% v/v | Used during U-¹³C₆ glucose tracing |
| Methanol | Metabolite extraction | Thermo Fisher Scientific; A456 | Pre-chilled at −80 °C | 100% | 80% v/v | Used for rapid metabolic quenching and extraction |
| Glucose | Seahorse glycolysis stress test | Agilent Technologies; 103020-100 | Seahorse assay medium | Prepared according to kit protocol | 10 mM final concentration | Acute injection during ECAR measurement |
| Oligomycin | Seahorse glycolysis stress test and mito stress test | Agilent Technologies; 103015-100 | Seahorse assay medium | Prepared according to kit protocol | 1.0 μM final concentration | Acute injection during ECAR or OCR measurement |
| FCCP | Seahorse mito stress test | Agilent Technologies; 103015-100 | Seahorse assay medium | Prepared according to kit protocol | 1.0 μM final concentration | Acute injection during OCR measurement |
| Rotenone/Antimycin A | Seahorse mito stress test | Agilent Technologies; 103015-100 | Seahorse assay medium | Prepared according to kit protocol | 0.5 μM each final concentration | Acute injection during OCR measurement |
| Lactate assay kit | Extracellular lactate measurement | Nanjing Jiancheng Bioengineering Institute; A019-2-1 | Provided in kit | Ready to use | According to kit protocol | Conditioned medium was collected and analyzed according to manufacturer’s instructions |
| HBS-EP+ running buffer | SPR assay | Cytiva; BR100669 | Ready to use | 1× | 1× | Used as SPR running buffer |
| CM5 sensor chip | SPR assay | Cytiva; BR100530 | Ready to use | Ready to use | Recombinant AARS1 immobilized to approximately 3,000 to 6,000 RU | Used for AARS1 immobilization |
| D-Luciferin | In vivo bioluminescence imaging | Gold Biotechnology; LUCK | Sterile PBS | 15 mg/mL | 150 mg/kg | Imaging performed 10 to 15 min after injection; In vivo: Intraperitoneal injection before IVIS imaging |
| ¹⁸F-FDG | PET/CT glucose uptake imaging | Local radiochemistry facility; Not applicable | Sterile injectable tracer | Prepared by facility | 7.4 to 11.1 MBq per mouse | PET/CT performed after 60 min uptake period; In vivo: Tail-vein injection |
| Isoflurane | Animal anesthesia | RWD Life Science; R510-22 | Vaporized anesthetic | Ready to use | 1.5% to 2.5% for maintenance | Used during imaging and procedures; In vivo: Inhalation anesthesia |
| DAB substrate kit | IHC chromogenic detection | Vector Laboratories; SK-4100 | Provided in kit | Ready to use | According to kit protocol | Color development monitored microscopically |
| BSA | Blocking and protein stabilization | Sigma-Aldrich; A7906 | PBS or TBST | 5% w/v | 5% for blocking; 0.1% for IGF-1 preparation | 30 to 60 min blocking or reagent preparation |
| Paraformaldehyde | Cell fixation | Electron Microscopy Sciences; 15710 | PBS | 16% stock | 4% | 15 to 30 min fixation at room temperature |
| Triton X-100 | Cell permeabilization | Sigma-Aldrich; T8787 | PBS | 10% stock | 0.1% to 0.3% | 10 to 15 min permeabilization |
| DAPI | Nuclear counterstaining | Thermo Fisher Scientific; D1306 | PBS | 1 mg/mL | 0.5 to 1 μg/mL | 5 to 10 min staining |

**Supplementary Table S5. Primer sequences used for RT-qPCR.**

| Gene | Forward primer (5'–3') | Reverse primer (5'–3') |
| --- | --- | --- |
| AARS1 | GTGAAGGTGGATGACAGCAGTG | CCACTTTCAGGTCACCGTAGATG |
| HK2 | GAGTTTGACCTGGATGTGGTTGC | CCTCCATGTAGCAGGCATTGCT |
| β-actin | GTGACGTTGACATCCGTAAAGA | GCCGGACTCATCGTACTCC |

**Supplementary Table S6. Primer sequences used for Chip-qPCR.**

| Gene | Forward primer (5'–3') | Reverse primer (5'–3') |
| --- | --- | --- |
| HK2 promoter | CAACATCGTGTCACCCAGCT | GCTAACTTCGGCCACAGGAT |
| HK2 −2 kb | CCCGGCATCCCTTGAATTCT | TCCAGGCCTGTCTCCAACTC |
| HK2 +2 kb | ATGTAGTGATGGCGCGTGAA | CAGAGCCACATCCCAGGAATT |
