# Supplementary material for: AARS1‐Mediated H3K27 Lactylation Rewires Glycolysis to Sustain Aggressive and Recurrent Bladder Cancer
Source: Adv Sci (Weinh). 2026 Aug 30:e77479. Online ahead of print. doi: 10.1002/advs.77479 (PMC13526531; doi:10.1002/advs.77479)
Supplement: Supplementary file 2 [file ADVS-9999-e77479-s002.docx]

**Figure S1. AARS1 is upregulated in bladder cancer and enriched in recurrent disease across public datasets and an independent patient cohort.** (a) AARS1 mRNA expression in TCGA-BLCA comparing normal bladder tissues and tumors (log₂[TPM+1]). (b) AARS1 mRNA expression in TCGA-BLCA comparing adjacent normal tissues and matched tumor tissues (log₂[TPM+1]). (c) Validation of elevated AARS1 mRNA expression in an independent GEO cohort (GSE3167) (normalized intensity). (d) Immunoblot analysis of AARS1 protein in freshly collected paired adjacent normal and tumor tissues from bladder cancer patients (n = 7 pairs), with β-actin as a loading control; right, densitometric quantification of relative AARS1 protein levels. (e) Immunoblot analysis of AARS1 protein in tumor tissues from non-recurrent and recurrent bladder cancer patients (n = 7 per group), with β-actin as a loading control. (f) Densitometric quantification of AARS1 protein levels in panel (e). (g) Kaplan–Meier survival analyses showing overall survival (left) and progression-free survival (right) stratified by AARS1 expression (high vs low, defined by the cohort median); *P* values were calculated by the log-rank test. For box/dot plots, each dot represents an individual sample; center lines indicate mean values. Two-group comparisons were assessed using two-sided Student’s *t* test.

**Figure S2 Establishment and validation of stable AARS1 gain- and loss-of-function models and their effects on bladder cancer cell proliferation and cell-cycle progression.** (a) RT–qPCR analysis of AARS1 mRNA levels in T24 and UMUC3 cells transduced with lentiviral AARS1 overexpression (LV-oe-AARS1), AARS1 knockdown (LV-sh-AARS1), or corresponding control vector (LV-vector/LV-ctrl). (b) Immunoblot validation of AARS1 protein expression in T24 and UMUC3 stable cell lines described in (a), with β-actin as a loading control; relative band intensities are shown below. (c,d) Representative immunofluorescence images showing AARS1 staining (red) in T24 (c) and UMUC3 (d) stable cell lines, with DAPI (blue) nuclear counterstaining; right, quantification of AARS1 immunofluorescence intensity (IOD/area). Scale bars, 100 μm. (e,f) CCK-8 assays showing cell viability of T24 (e) and UMUC3 (f) stable cell lines over the indicated time course. (g,h) Colony formation assays in T24 (g) and UMUC3 (h) stable cell lines, with quantification of colony numbers. (i) EdU incorporation assays in UMUC3 stable cell lines; representative images and quantification of EdU-positive cells are shown. Scale bars, 100 μm. (j) Flow cytometric analysis of cell-cycle distribution in T24 and UMUC3 stable cell lines; representative DNA-content histograms are shown (left), with quantification of the G2/M fraction (right). Data are presented as mean ± s.d. from at least three independent experiments unless otherwise stated. *P* values were determined by two-sided Student’s *t* test (for two-group comparisons) or one-way ANOVA with appropriate post hoc tests (for multiple groups).

**Figure S3 AARS1 promotes migration/invasion, suppresses apoptosis, and reinforces EMT programs in bladder cancer cells and in vivo.** (a,b) Wound-healing assays in T24 (a) and UMUC3 (b) cells stably expressing LV-oe-AARS1, LV-ctrl, or LV-sh-AARS1. Representative images at 0 h and 24 h are shown (left), with quantification of wound-healing rate (right). (c,d) Matrigel-coated Transwell invasion assays in T24 and UMUC3 cells with the indicated AARS1 manipulations. Representative invaded cells are shown in (c), and quantification of invaded cells per field is shown in (d). (e) TUNEL staining in UMUC3 cells with LV-oe-AARS1, LV-ctrl, or LV-sh-AARS1. Representative images (left) and quantification of TUNEL-positive cells (right) are shown. Scale bars, 100 μm. (f) Flow cytometric analysis of apoptosis by Annexin V/7-AAD staining in T24 and UMUC3 cells with the indicated AARS1 manipulations. Representative dot plots (left) and quantification of apoptotic rate (right) are shown. (g,h) Immunoblot analysis of EMT-associated markers (E-cadherin, N-cadherin, Vimentin, and SNAIL2) in T24 and UMUC3 cells after AARS1 knockdown (g) or AARS1 overexpression (h), with β-actin as a loading control. Relative band intensities are indicated by dot plots. (i) Immunofluorescence staining of E-cadherin (red) and N-cadherin (cyan) in UMUC3 cells with LV-oe-AARS1, LV-ctrl, or LV-sh-AARS1. Representative images (left) and quantification (IOD/area; right) are shown. DAPI (blue) marks nuclei. Scale bars, 100 μm. (j) Quantification of IHC signals (IOD/area) for AARS1 and Ki67 (left), and for E-cadherin and N-cadherin (right) in xenograft tumors derived from cells with AARS1 overexpression or knockdown, as indicated. (k) Representative images of lungs from the tail-vein metastasis model using luciferase-labeled cells with LV-oe-AARS1, LV-ctrl, or LV-sh-AARS1. (l,m) Body weight (l) and subcutaneous tumor growth curves (m) of mice bearing xenografts derived from cells with LV-oe-AARS1, LV-ctrl, or LV-sh-AARS1. (n,o) Quantification of lung metastatic burden, including the number of metastatic nodules per lung (n) and the percentage of lung volume occupied by tumor lesions (o). Data are presented as mean ± s.d. from at least three independent experiments unless otherwise stated. Statistical significance was determined by two-sided Student’s *t* test (two groups) or one-way ANOVA with appropriate post hoc tests (multiple groups).

**Figure S4 The pro-tumorigenic functions of AARS1 depend on its enzymatic integrity.** (a,b) CCK-8 assays showing cell viability over time in T24 (a) and UMUC3 (b) cells transduced with control lentivirus (LV-ctrl), AARS1 knockdown (LV-sh-AARS1), or shAARS1 cells rescued with AARS1 wild type (AARS1-WT) or the AARS1-5M mutant (M46A/R77A/N216A/D239A/G241A). (c,d) Colony formation assays and quantification of colony numbers in T24 (c) and UMUC3 (d) cells with the indicated genetic manipulations. (e,f) EdU incorporation assays in T24 (e) and UMUC3 (f) cells with the indicated constructs. Representative immunofluorescence images (DAPI, blue; EdU, red) and quantification of EdU-positive cells are shown. Scale bars, 100 μm. (g) Cell-cycle profiling by flow cytometry (PI staining) in T24 and UMUC3 cells with the indicated manipulations, with quantification of the G2/M fraction. (h–j) Wound-healing assays assessing migratory capacity. Representative images at 0 h and 24 h in T24 (h) and UMUC3 (j) cells are shown, with corresponding quantification of wound-healing rate in T24 (i) and UMUC3 (j, right). (k,l) Transwell migration assays in T24 and UMUC3 cells with representative images (k) and quantification of migrated cells per field (l). (m) Matrigel-coated Transwell invasion assays in T24 and UMUC3 cells with representative images (left) and quantification of invaded cells per field (right). Scale bars, 100 μm. Data are presented as mean ± s.d. from at least three independent experiments unless otherwise indicated. Statistical significance was assessed by two-sided Student’s *t* test (two groups) or one-way ANOVA with appropriate post hoc tests (multiple groups).

**Figure S5 AARS1 enzymatic integrity is required for apoptosis suppression and EMT remodeling.** (a) TUNEL staining in T24 cells transduced with LV-ctrl, LV-sh-AARS1, or shAARS1 cells rescued with AARS1-WT or the AARS1-5M mutant (M46A/R77A/N216A/D239A/G241A). Representative images (DAPI, blue; TUNEL, red) and quantification of TUNEL-positive cells are shown. Scale bars, 100 μm. (b) Representative flow cytometry plots (Annexin V–APC/7-AAD) in T24 and UMUC3 cells with the indicated manipulations, with quantification of apoptotic rates in T24. (c) Quantification of apoptotic rates in UMUC3 cells measured by Annexin V–APC/7-AAD staining. (d,e) Immunoblotting of AARS1 and EMT markers (E-cadherin, N-cadherin, Vimentin, SNAIL2) in T24 (d) and UMUC3 (e) cells across the indicated genetic conditions. β-actin served as a loading control. Dot plots summarize relative band intensities normalized to β-actin. (f) Immunofluorescence staining of E-cadherin (red) and N-cadherin (cyan) with DAPI counterstain (blue) in T24 and UMUC3 cells under the indicated conditions, with quantification of fluorescence intensity (IOD/area). Scale bars, 100 μm. Data are presented as mean ± s.d. from at least three independent experiments unless otherwise stated. Statistical significance was assessed by two-sided Student’s *t* test (two groups) or one-way ANOVA with appropriate post hoc testing (multiple groups).

**Figure S6 AARS1 regulates PI3K–AKT–mTOR signaling in bladder cancer cells.** (a,b) Immunoblot analysis of PI3K–AKT–mTOR pathway activation in T24 (a) and UMUC3 (b) cells following AARS1 knockdown (LV-sh-AARS1) versus control (LV-ctrl). Phosphorylated PI3K p85 (Tyr458), AKT (Ser473), mTOR (Ser2448), and S6K (Thr389) and their corresponding total proteins are shown; β-actin served as the loading control. Dot plots summarize relative band intensities normalized to β-actin and expressed relative to the corresponding control. (c) Immunoblot analysis of PI3K–AKT–mTOR signaling in T24 cells with AARS1 overexpression (LV-oe-AARS1) or matched control (LV-ctrl), with quantification shown as dot plots (normalized to β-actin). (d) Independent validation of AARS1 knockdown–associated suppression of PI3K–AKT–mTOR signaling in UMUC3 cells (representative immunoblots and corresponding densitometric dot plots). (e,f) Pharmacologic epistasis analysis showing that PI3K inhibition by wortmannin attenuates AARS1 overexpression–induced pathway activation, whereas IGF-1 partially restores pathway signaling in AARS1-silenced cells in T24 (e) and UMUC3 (f). Representative immunoblots are shown with densitometric dot-plot summaries normalized to β-actin. All immunoblots are representative of at least three independent experiments with similar results.

**Figure S7. AARS1 is associated with receptor-proximal PI3K–AKT signaling output in bladder cancer cells.** (a-b) Representative immunoblots showing p-EGFR, EGFR, p-IGF1Rβ, IGF1Rβ, p-IRS1, IRS1, p-AKT, and AKT levels in T24 and UMUC3 cells with AARS1 knockdown or AARS1 overexpression, with or without Wortmannin or IGF-1 treatment. β-actin was used as a loading control. Data are representative of three independent experiments.

**Figure S8 Pharmacologic epistasis supports a PI3K-dependent role of AARS1 in driving proliferation and cell-cycle progression.** (a,b) CCK-8 assays showing cell viability over time in T24 (a) and UMUC3 (b) cells with AARS1 overexpression (LV-oe-AARS1) or AARS1 knockdown (LV-sh-AARS1), and the indicated pharmacologic modulation with wortmannin (PI3K inhibitor) or IGF-1. (c,d) Colony formation assays in T24 (c) and UMUC3 (d) cells under the indicated genetic (LV-oe-AARS1, LV-sh-AARS1, and matched LV-ctrl) and pharmacologic (wortmannin or IGF-1) conditions, with quantification of colony numbers. (e) Representative EdU incorporation images (DAPI, blue; EdU, red) and quantification of EdU-positive cells in UMUC3 cells treated as indicated, showing that wortmannin dampens AARS1 overexpression–driven DNA synthesis, whereas IGF-1 partially rescues the proliferative defect caused by AARS1 silencing. (f) Representative flow-cytometry histograms of PI staining (DNA content) illustrating cell-cycle distributions in T24 and UMUC3 cells under the indicated genetic and pharmacologic conditions. (g,h) Quantification of the G2/M-phase fraction in T24 (g) and UMUC3 (h) cells corresponding to (f). Data are shown as mean ± s.d. (where applicable). Each dot represents an independent replicate. P values are indicated in the panels. All experiments were repeated at least three times with similar results.

**Figure S9 PI3K signaling mediates the pro-migratory/invasive and anti-apoptotic effects of AARS1.** (a,b) Representative wound-healing images at 0 h and 24 h in T24 (a) and UMUC3 (b) cells with AARS1 overexpression (LV-oe-AARS1) or AARS1 knockdown (LV-sh-AARS1), treated with wortmannin or IGF-1 as indicated. Right, quantification of wound closure. (c) Quantification of Transwell migration in UMUC3 cells under the indicated genetic (LV-oe-AARS1, LV-sh-AARS1, and matched LV-ctrl) and pharmacologic (wortmannin or IGF-1) conditions. (d) Representative images of Matrigel invasion assays in T24 and UMUC3 cells with the indicated genetic and pharmacologic treatments. Right, quantification of invaded cells per field. (e) Representative TUNEL staining images (DAPI, blue; TUNEL, red) in UMUC3 cells treated as indicated, with quantification of TUNEL-positive cells. (f) Representative flow-cytometry plots of Annexin V/7-AAD staining in T24 and UMUC3 cells under the indicated conditions. (g) Quantification of apoptotic rates derived from (f) in T24 and UMUC3 cells. Data are presented as mean ± s.d. (where applicable). Each dot represents an independent replicate. P values are indicated in the panels. Experiments were repeated at least three times with similar results.

**Figure S10 PI3K signaling is required for AARS1-driven EMT remodeling and metastatic progression in vitro and in vivo.** (a,b) Immunoblot analysis of EMT markers (E-cadherin, N-cadherin, vimentin and SNAIL2) in T24 (a) and UMUC3 (b) cells with AARS1 overexpression (LV-oe-AARS1) or AARS1 knockdown (LV-sh-AARS1), treated with wortmannin or IGF-1 as indicated. Right, corresponding densitometric summary shown as dot heatmaps normalized to β-actin. (c,d) Representative immunofluorescence images of E-cadherin (red) and N-cadherin (cyan) with DAPI nuclear counterstain (blue) in T24 (c) and UMUC3 (d) cells under the indicated genetic and pharmacologic conditions. Bottom, quantification of fluorescence intensity (IOD/area). (e,f) Body weight (e) and subcutaneous xenograft growth curves (f) of mice bearing tumors derived from the indicated T24 cell groups with wortmannin treatment for AARS1-overexpressing tumors or IGF-1 treatment for AARS1-silenced tumors, as indicated. (g,h) Quantification of lung metastatic burden in the tail-vein model, including the number of visible metastatic nodules per lung (g) and the percentage of lung area occupied by tumor lesions (h), across the indicated treatment groups. (i) Representative IHC staining of xenograft tumors for Ki67, N-cadherin and E-cadherin, with quantification of staining intensity (IOD/area) shown on the right. Data are presented as mean ± s.d. (where applicable). Each dot represents an individual biological replicate (cells) or an individual mouse (in vivo). P values are indicated in the panels. Experiments were repeated independently with similar results.

**Figure S11 AARS1 preferentially enhances glycolysis-to-lactate flux with minimal effects on mitochondrial respiration.** (a,b) Seahorse glycolysis stress test in UMUC3 cells with AARS1 overexpression (LV-oe-AARS1) or AARS1 knockdown (LV-sh-AARS1), with pharmacologic epistasis using wortmannin (PI3K inhibitor) or IGF-1 (PI3K pathway agonist) as indicated. (a) Representative ECAR traces following sequential injection of glucose, oligomycin and 2-deoxy-D-glucose (2-DG). (b) Quantification of glycolysis and glycolytic capacity derived from ECAR profiles. (c) Lactate levels in culture supernatants under the indicated conditions. (d) ^13C-glucose tracing showing fractional enrichment of lactate (M+3) in UMUC3 cells, indicating altered glucose-to-lactate carbon flux upon AARS1 manipulation and PI3K pathway modulation. (e–j) ^13C-glucose tracing of TCA-related metabolites showing fractional enrichment of acetyl-CoA (M+2), citrate (M+2) and malate (M+2) in T24 (e–g) and UMUC3 (h–j) cells under the indicated conditions, demonstrating no significant redistribution of glucose-derived carbon into the TCA cycle. (k,l) Seahorse mitochondrial stress tests in T24 (k) and UMUC3 (l) cells. Left, representative OCR traces following sequential injection of oligomycin, FCCP and rotenone/antimycin A (Rot/AA). Right, quantification of basal respiration, ATP-linked respiration, maximal respiration and spare respiratory capacity. Data are presented as mean ± s.d. (where applicable). Each dot represents an independent biological replicate. Statistical significance is indicated in the panels; ns, not significant. Experiments were repeated independently with similar results.

**Figure S12 Lactate rescues PI3K blockade–induced suppression of AARS1-driven proliferation, supporting a lactate-dependent downstream program.** (a,b) Cell viability (CCK-8) time-course assays in T24 (a) and UMUC3 (b) cells under the indicated genetic and pharmacologic conditions: AARS1 overexpression with PI3K inhibition (wortmannin) with or without exogenous lactate supplementation, and AARS1 knockdown with IGF-1 stimulation with or without glycolysis blockade by 2-deoxy-D-glucose (2-DG). (c,d) Colony formation assays in T24 (c) and UMUC3 (d) cells treated as indicated. Quantification of colony numbers is shown. (e) EdU incorporation assay in UMUC3 cells under the indicated conditions. Representative images (DAPI, EdU) and quantification of EdU-positive cells are shown. (f) Cell-cycle analysis by PI staining and flow cytometry in T24 and UMUC3 cells under the indicated conditions. Representative DNA content histograms and quantification of G2/M-phase fractions are shown. Data are presented as mean ± s.d. . Each dot represents an independent biological replicate. Statistical significance is indicated in the panels. Experiments were repeated independently with similar results.

**Figure S13 Lactate reinstates AARS1-dependent motility and survival programs downstream of PI3K signaling.** (a) Representative wound-healing images (0 and 24 h) of T24 and UMUC3 cells under the indicated genetic manipulations (LV-oe-AARS1 or LV-sh-AARS1) and treatments, including PI3K inhibition (wortmannin), pathway activation (IGF-1), lactate supplementation, and glycolysis blockade (2-DG). (b) Quantification of wound closure rates for T24 cells under the indicated conditions. (c) Transwell migration assay in UMUC3 cells. Quantification of migrated cell numbers is shown. (d) Matrigel-coated Transwell invasion assays in T24 and UMUC3 cells. Representative images and quantification of invaded cells are shown. (e,f) TUNEL staining in T24 (e, quantification) and UMUC3 (f, representative images and quantification) cells treated as indicated. Nuclei were counterstained with DAPI. (g) Flow cytometric analysis of apoptosis using Annexin V/7-AAD staining in T24 and UMUC3 cells under the indicated conditions. Representative plots are shown. (h) Summary quantification of apoptotic rates (Annexin V–positive cells) for T24 and UMUC3 under the indicated conditions, highlighting reversal by lactate and suppression by 2-DG. Data are presented as mean ± s.d. . Each dot represents an independent biological replicate. Statistical significance is indicated in the panels. Experiments were repeated independently with similar results.

**Figure S14 Metabolic gating of EMT and tumor growth downstream of the AARS1–PI3K axis in bladder cancer.** (a,b) Immunoblot analysis of EMT markers (E-cadherin, N-cadherin, Vimentin and SNAI2) in T24 (a) and UMUC3 (b) cells subjected to AARS1 overexpression or knockdown, with pharmacologic modulation as indicated (wortmannin, IGF-1, lactate supplementation and 2-DG). β-actin served as a loading control. Dot plots beside blots denote relative band intensities. (c) Representative immunofluorescence images of E-cadherin (red) and N-cadherin (cyan) in UMUC3 cells under the indicated conditions, with DAPI nuclear counterstaining (blue). Right, quantification of fluorescence intensity (IOD/area) for E-cadherin and N-cadherin. (d) Representative images of excised xenograft tumors from the indicated treatment/genetic groups. (e) Tumor weights at endpoint from the xenograft experiments shown in (d). (f,g) Tumor growth curves (f) and body weight monitoring (g) for mice bearing subcutaneous tumors under the indicated conditions. (h) Representative immunohistochemistry (IHC) staining of Ki67, N-cadherin and E-cadherin in xenograft tumors from the indicated groups, with quantification of IHC signals (IOD/area). (i) Quantification of tumor glucose uptake from ¹⁸F-FDG PET/CT imaging (SUVmean) in the indicated groups. (j,k) Quantification of lung metastatic burden in the tail-vein metastasis model, shown as the number of metastatic lung nodules (j) and the percentage of lung area occupied by tumor (k) across groups. Data are presented as mean ± s.d. . Each dot represents an independent biological replicate (in vitro) or an individual mouse (in vivo). Statistical significance is indicated in the panels. Experiments were repeated independently with similar results.

**Figure S15 AARS1 regulates global protein lactylation and histone lactylation in bladder cancer cells in a PI3K–glycolysis–lactate–dependent manner.** (a,b) Immunoblot analysis of pan-lactylation in T24 and UMUC3 cells following AARS1 knockdown (a) or AARS1 overexpression (b). β-actin served as a loading control. Dot plots indicate relative band intensities. (c) Pan-lactylation immunoblot in UMUC3 cells under the indicated genetic manipulations (LV-oe-AARS1 or LV-sh-AARS1) combined with pathway modulation (wortmannin and/or IGF-1). β-actin served as a loading control; relative band intensities are shown below. (d,e) Pan-lactylation immunoblot in T24 (d) and UMUC3 (e) cells across the indicated epistasis conditions, including PI3K inhibition (wortmannin), growth-factor stimulation (IGF-1), lactate supplementation, and glycolysis blockade (2-DG). β-actin served as a loading control; relative band intensities are shown below. (f-g) Representative immunoblots and densitometric quantification of multiple histone lactylation marks in T24 and UMUC3 cells with AARS1 knockdown or overexpression. The examined marks included H3K23la, H3K27la, H3K56la, H3K9la, H4K12la, H4K5la, H4K16la, H3K14la, and H3K18la, with total H3 and H4 as loading controls. Dot plots denote relative band intensities. Experiments were repeated independently with similar results.

**Figure S16 AARS1 controls H3K27 lactylation and global lactylation in bladder cancer cells through PI3K signaling and glycolysis-derived lactate.** (a,b) Immunoblot analysis of H3K27la in T24 (a) and UMUC3 (b) cells under the indicated genetic perturbations (LV-oe-AARS1 or LV-sh-AARS1) combined with PI3K inhibition (wortmannin) or pathway reactivation (IGF-1). Total H3 served as a histone loading control. Dot plots indicate relative band intensities. (c,d) Immunoblot analysis of H3K27la in T24 (c) and UMUC3 (d) cells following epistasis modulation of the AARS1–PI3K–glycolysis axis, including wortmannin, IGF-1, exogenous lactate supplementation, and glycolysis blockade (2-DG). Total H3 served as a loading control; relative band intensities are shown below. (e) Quantification of immunofluorescence signal intensities (IOD/area) for pan-lactylation (top) and H3K27la (bottom) in T24 cells across the indicated conditions. (f) Representative immunofluorescence images of pan-lactylation and H3K27la in UMUC3 cells under the indicated conditions, with corresponding IOD/area quantification shown at right. Nuclei were counterstained with DAPI. (g,h) Representative immunofluorescence images of pan-lactylation and H3K27la in T24 (g) and UMUC3 (h) cells following combined genetic and pharmacologic modulation (wortmannin, IGF-1, lactate, and 2-DG), as indicated. (i,j) Quantification of pan-lactylation and H3K27la immunofluorescence signals (IOD/area) in T24 (i) and UMUC3 (j) cells for the conditions shown in (g,h). Data are presented as mean ± SD from independent experiments; statistical significance is indicated on the plots.

**Figure S17 AARS1 promotes HK2 transcription and expression through PI3K signaling, thereby reinforcing the glycolytic program in bladder cancer cells.** (a) ChIP–qPCR analysis of HK2 promoter occupancy in UMUC3 cells with AARS1 overexpression (LV-oe-AARS1) or knockdown (LV-sh-AARS1). IgG served as a negative control, and enrichment is shown as fold over IgG. (b,c) ChIP–qPCR analysis of HK2 promoter occupancy in T24 (b) and UMUC3 (c) cells under the indicated epistasis conditions, including PI3K inhibition (wortmannin) and pathway activation (IGF-1) in AARS1 gain- or loss-of-function backgrounds. (d,e) ChIP–qPCR analysis of HK2 promoter occupancy in T24 (d) and UMUC3 (e) cells following metabolic rescue or blockade, including lactate supplementation and glycolysis inhibition (2-DG), as indicated. (f) Immunoblot analysis of HK2 protein levels in T24 and UMUC3 cells after AARS1 knockdown; β-actin served as a loading control. Dot plots indicate relative band intensities. (g) Immunoblot analysis of HK2 protein levels in T24 and UMUC3 cells after AARS1 overexpression; β-actin served as a loading control, with corresponding relative intensity quantification. (h) Immunoblot analysis of HK2 expression in UMUC3 cells subjected to combined genetic modulation of AARS1 and PI3K pathway perturbation (wortmannin) or activation (IGF-1), as indicated; β-actin served as a loading control. (i,j) Representative immunofluorescence staining of HK2 in T24 (i) and UMUC3 (j) cells under the indicated conditions, with quantification of fluorescence intensity (IOD/area) shown at right. Nuclei were counterstained with DAPI. (k,l) Immunoblot analysis of HK2 protein levels in T24 (k) and UMUC3 (l) cells following integrated genetic, signaling, and metabolic modulation (wortmannin, IGF-1, lactate, and 2-DG), as indicated; β-actin served as a loading control. Data are presented as mean ± SD from independent experiments; statistical significance is indicated on the plots.

**Figure S18 HK2 is upregulated in bladder cancer tissues and cell lines and associates with tumor recurrence.** (a) Immunoblot analysis of HK2 protein levels in paired bladder cancer tissues and adjacent normal tissues (n = 7 pairs). β-actin served as a loading control. Dot plots indicate the relative band intensities of HK2 normalized to β-actin. (b) Immunoblot analysis of HK2 expression in bladder cancer tissues from non-recurrent and recurrent cases (paired samples from the same patients; n = 7 pairs). β-actin served as a loading control, with relative HK2/β-actin band intensity quantification shown below. (c) Immunoblot analysis of HK2 protein levels in a normal urothelial cell line (SV-HUC-1) and bladder cancer cell lines (J82, T24, TCCSUP, 5637, and UMUC3). β-actin served as a loading control; dot plots show relative HK2/β-actin intensities. (d) Representative immunofluorescence staining of HK2 in SV-HUC-1 and the indicated bladder cancer cell lines. Nuclei were counterstained with DAPI. Scale bar, 50 μm.

**Figure S19 Validation of HK2 manipulation and its effects on bladder cancer cell proliferation and cell-cycle progression.** (a) RT–qPCR analysis of HK2 mRNA levels in T24 and UMUC3 cells transduced with lentiviral control (LV-ctrl), HK2 knockdown (LV-sh-HK2), or HK2 overexpression (LV-oe-HK2). (b,c) Immunoblot verification of HK2 knockdown (b) and HK2 overexpression (c) in T24 and UMUC3 cells. β-actin served as a loading control; dot plots indicate relative HK2/β-actin band intensities. (d,e) Representative immunofluorescence staining of HK2 in T24 (d) and UMUC3 (e) cells under the indicated conditions, with quantitative analysis shown on the right. Nuclei were counterstained with DAPI. Scale bar, 50 μm. (f,g) CCK-8 assays showing cell viability/proliferation curves of T24 (f) and UMUC3 (g) cells with HK2 overexpression or knockdown. (h) Colony formation assays in T24 and UMUC3 cells following HK2 manipulation, with representative images (left) and quantification of colony numbers (right). (i–k) EdU incorporation assays in T24 (i,j) and UMUC3 (k) cells with HK2 overexpression or knockdown. Representative images are shown in (i) and (k), with quantification of EdU-positive cells in (j) and (k). Nuclei were counterstained with DAPI. Scale bar, 50 μm. (l) Flow cytometric analysis of cell-cycle distribution (PI staining) in T24 and UMUC3 cells under the indicated conditions, with quantification of the G2/M fraction shown on the right. Data are presented as mean ± SD from at least three independent experiments. Statistical significance was determined by two-tailed Student’s *t*-test or one-way ANOVA as appropriate; *P* values are indicated in the panels.

**Figure S20 HK2 promotes bladder cancer cell migration/invasion and EMT while suppressing apoptosis.** (a,b) Wound-healing assays in T24 (a) and UMUC3 (b) cells transduced with LV-oe-HK2, LV-ctrl, or LV-sh-HK2. Representative images at 0 h and 24 h and quantification of wound-healing rate are shown. (c,d) Transwell migration assays in T24 and UMUC3 cells with HK2 overexpression or knockdown. Representative images are shown in (c), with additional representative fields in (d). (e,f) Quantification of migrated (e) and invaded (f) cells per field for the indicated groups in T24 and UMUC3 cells. (g–i) TUNEL staining of T24 (g) and UMUC3 (h) cells under the indicated conditions, with quantification of TUNEL-positive cells shown in (i). Nuclei were counterstained with DAPI. Scale bar, 50 μm. (j) Flow cytometric analysis of apoptosis by Annexin V-APC/7-AAD staining in T24 and UMUC3 cells with HK2 overexpression or knockdown, with quantification shown on the right. (k,l) Immunoblot analysis of EMT-associated markers (E-cadherin, N-cadherin, Vimentin and SNAI2) in T24 and UMUC3 cells following HK2 knockdown (k) or HK2 overexpression (l). β-actin served as a loading control; dot plots indicate relative band intensities. (m,n) Representative immunofluorescence staining of E-cadherin and N-cadherin in T24 (m) and UMUC3 (n) cells with HK2 overexpression or knockdown, with quantitative analysis shown on the right. Nuclei were counterstained with DAPI. Scale bar, 50 μm. Data are presented as mean ± SD from at least three independent experiments. Statistical significance was determined by two-tailed Student’s *t*-test or one-way ANOVA as appropriate; *P* values are indicated in the panels.

**Figure S21 HK2 contributes to AARS1-associated lactate production and lactylation output.** (a,b) Extracellular lactate levels in T24 (a) and UMUC3 (b) cells with AARS1 knockdown or overexpression combined with HK2 re-expression or HK2 silencing. Lactate levels were normalized to total protein content. (c,d) Representative immunoblots and densitometric quantification of pan-lactylation in T24 (c) and UMUC3 (d) cells with the indicated genetic manipulations. β-actin was used as a loading control. (e,f) Representative immunoblots and densitometric quantification of H3K27la in T24 (e) and UMUC3 (f) cells with the indicated genetic manipulations. Total H3 was used as a loading control. Data are presented as mean ± SD from three independent experiments. Statistical significance was determined by one-way ANOVA followed by Tukey’s post hoc test. P values are indicated in the panels.

**Figure S22 HK2 promotes bladder cancer tumor growth and lung metastasis in vivo.** (a) Representative images of subcutaneous xenograft tumors derived from T24/UMUC3 cells stably transduced with LV-oe-HK2, LV-ctrl, or LV-sh-HK2 (left), with quantification of tumor weight at endpoint (right). (b) Tumor growth curves showing tumor volume measured over time in the indicated groups. (c) Representative images of mice bearing xenograft tumors at the experimental endpoint. (d) Body-weight monitoring of mice during the study. (e) Immunohistochemistry (IHC) staining of HK2, Ki67, N-cadherin and E-cadherin in xenograft tumors, with IOD/area quantification shown on the right. (f) Representative images of lungs and H&E staining from the experimental metastasis model (left), with quantification of lung metastatic nodules (middle) and lung tumor burden (right) in the indicated groups. Data are presented as mean ± SD; each dot represents one mouse. Statistical significance is indicated by the P values shown in the panels.

**Figure S23 HK2 mediates AARS1-driven malignant phenotypes in bladder cancer cells.** (a,b) CCK-8 assays showing cell viability of T24 (a) and UMUC3 (b) cells under the indicated conditions (LV-oe-AARS1, LV-sh-AARS1, and corresponding controls), with HK2 silencing (si-HK2) in AARS1-overexpressing cells or HK2 overexpression (oe-HK2) in AARS1-depleted cells. (c,d) EdU incorporation assays evaluating DNA synthesis. Quantification of EdU-positive cells in T24 and UMUC3 (c) and representative EdU staining images in UMUC3 (d) (DAPI, blue; EdU, red). (e-g) Cell-cycle distribution analyzed by PI staining and flow cytometry. Representative histograms are shown in (e), with quantification of the G2/M fraction in T24 (f) and UMUC3 (g). (h) Transwell migration assays with quantification of migrated cells in T24 and UMUC3. (i,j) Matrigel invasion assays. Representative images and quantification in T24 (i) and quantification in UMUC3 (j). (k,l) Apoptosis analyses by TUNEL staining and flow cytometry. Representative TUNEL staining images in UMUC3 (k) (DAPI, blue; TUNEL, red) and quantification of TUNEL-positive cells (UMUC3) and apoptosis rates (T24 and UMUC3) (l). (m) Representative Annexin V-APC/7-AAD flow cytometry plots for apoptosis assessment in T24 and UMUC3 under the indicated conditions. (n) Immunofluorescence staining of EMT markers in UMUC3 cells (E-cadherin, red; N-cadherin, cyan; DAPI, blue) with quantification (IOD/area). Data are presented as mean ± SD; P values are indicated in the panels.

**Figure S24 HK2 mediates AARS1-driven tumor growth, glycolytic activity, and lung metastatic burden in vivo.** (a) Xenograft tumor weights from mice implanted with bladder cancer cells bearing the indicated manipulations (LV-oe-AARS1, LV-oe-AARS1 + si-HK2, LV-ctrl, LV-sh-AARS1 + oe-HK2, and LV-sh-AARS1). (b) Xenograft growth curves showing tumor volumes measured over time. (c) Representative images of mice at endpoint and body weight monitoring throughout the experiment. (d) IHC staining of HK2, Ki67, N-cadherin, and E-cadherin in xenograft tumors, with quantification (IOD/area). (e) Quantification of tumor glucose uptake presented as SUVmean (as indicated). (f) Quantification of lung metastatic nodules per lung. (g) Quantification of lung tumor burden (tumor occupancy of lung volume). Data are presented as mean ± SD; each dot represents one mouse. P values are indicated.

**Figure S25 Eltrombopag suppresses AARS1-driven PI3K–AKT–mTOR signaling in bladder cancer cells.** (a) Immunoblot/quantification heatmap showing p-PI3K p85 (Tyr458), PI3K p85α, p-AKT (Ser473), AKT, p-mTOR (Ser2448), mTOR, p-S6K (Thr389), S6K, and β-actin in T24 cells transduced with lentiviral control (LV-ctrl), AARS1 knockdown (LV-sh-AARS1), or AARS1 overexpression (LV-oe-AARS1), followed by eltrombopag or vehicle (DMSO) treatment as indicated. (b) Representative western blots of the indicated proteins in UMUC3 cells under the same treatments. (c) Densitometric summary/heatmap for UMUC3 blots. β-actin served as a loading control.

**Figure S26 Eltrombopag attenuates AARS1-associated glycolytic reprogramming and lactate output.** (a) Seahorse XF glycolysis stress test (ECAR) in UMUC3 cells with LV-oe-AARS1, LV-sh-AARS1, or LV-ctrl treated with eltrombopag or DMSO; injection points (glucose, oligomycin, and 2-DG) are indicated. (b) Quantification of glycolysis and glycolytic capacity from (a). (c) ECAR profile during the mitochondrial stress test in T24 cells; injection points (oligomycin, FCCP, and rotenone/antimycin A) are indicated. (d) Seahorse XF mitochondrial stress test (OCR) trace in UMUC3 cells under the indicated conditions. (e,f) Quantification of basal respiration, ATP-linked respiration, maximal respiration, and spare respiratory capacity in T24 (e) and UMUC3 (f). (g) Lactate level/production in UMUC3 cells under the indicated treatments. (h–m) Stable-isotope tracing showing fractional enrichment (M+2) of acetyl-CoA, citrate, and malate in T24 (h–j) and UMUC3 (k–m) cells under the indicated conditions. Data are shown as mean ± SD; P values and “ns” (not significant) are indicated.

**Figure S27 Eltrombopag reduces global lactylation, H3K27 lactylation, and HK2 expression regulated by AARS1.** (a,b) Western blot analysis of pan-lactylation in T24 (a) and UMUC3 (b) cells transduced with LV-ctrl, LV-sh-AARS1, or LV-oe-AARS1 and treated with eltrombopag or DMSO as indicated; β-actin was used as a loading control. (c,d) Immunoblotting of H3K27la in T24 (c) and UMUC3 (d); total H3 served as a loading control. (e,f) Immunoblotting of HK2 in T24 (e) and UMUC3 (f) with β-actin as a loading control. Relative band intensities are summarized beneath the blots.

**Figure S28 Immunofluorescence validation of eltrombopag effects on lactylation and HK2.** (a,b) Representative immunofluorescence images of pan-lactylation and H3K27la in T24 (a) and UMUC3 (b) cells with AARS1 overexpression/knockdown and eltrombopag or DMSO treatment as indicated. Nuclei were counterstained with DAPI. (c,d) Quantification of fluorescence intensity (IOD/area) for H3K27la and pan-lactylation in T24 (c) and UMUC3 (d). (e) Representative HK2 immunofluorescence images (UMUC3) and quantification (T24 and UMUC3) under the indicated conditions. Scale bars are shown in images; each dot represents a biological replicate; P values are indicated.

**Figure S29 Eltrombopag counteracts AARS1-dependent proliferation and cell-cycle progression.** (a,b) CCK-8 cell viability curves in T24 (a) and UMUC3 (b) cells with LV-oe-AARS1 or LV-sh-AARS1 combined with HK2 knockdown (si-HK2) or HK2 overexpression (oe-HK2) as indicated. (c) EdU incorporation (percentage of EdU⁺ cells) in T24 and UMUC3 under the indicated rescue conditions. (d) Representative EdU staining images (UMUC3; DAPI, EdU, and merged). (e) Cell-cycle distribution (PI staining) with representative histograms and quantification of G2/M fraction. (f–h) Quantification of G2/M fraction and Transwell migration cell numbers in T24 and UMUC3 under the indicated conditions. (i,j) Representative Transwell invasion images and quantification of invaded cells in T24 (i) and UMUC3 (j). (k) Representative TUNEL staining images (UMUC3) and quantification of TUNEL⁺ cells. (l) Quantification of apoptosis rate under the indicated conditions. Data are shown as mean ± SD; each dot represents a biological replicate; P values are indicated.

**Figure S30 Eltrombopag suppresses AARS1-driven migration/invasion and promotes apoptosis.** (a,b) Wound-healing assay images (0 h and 24 h) and quantification in T24 (a) and UMUC3 (b) cells with AARS1 overexpression/knockdown treated with eltrombopag or DMSO as indicated. (c) Representative Transwell migration images. (d,e) Quantification of migrated cells per field in T24 (d) and UMUC3 (e). (f) Quantification of invaded cells per field (Transwell invasion) in T24 and UMUC3. (g,h) Representative TUNEL staining images in T24 (g) and UMUC3 (h). (i) Quantification of TUNEL⁺ cells in T24 and UMUC3. (j) Representative Annexin V/7-AAD flow cytometry plots and quantification of apoptosis rate in T24. (k) Quantification of apoptosis rate in UMUC3. Data are shown as mean ± SD; P values are indicated.

**Figure S31 Eltrombopag reverses AARS1-associated EMT marker changes.** (a,b) Western blot analysis of EMT markers (E-cadherin, N-cadherin, vimentin, and SNAI2) in T24 (a) and UMUC3 (b) cells with LV-oe-AARS1, LV-sh-AARS1, or LV-ctrl treated with eltrombopag or DMSO as indicated; β-actin served as a loading control. Relative band intensities are summarized as dot heatmaps. (c,d) Representative immunofluorescence co-staining of E-cadherin (red) and N-cadherin (green) with DAPI in T24 (c) and UMUC3 (d), with corresponding quantification (IOD/area). Data are shown as mean ± SD; P values are indicated.

**Figure S32 Eltrombopag inhibits AARS1-driven tumor growth and metastatic burden in vivo.** (a) Representative images of excised subcutaneous xenograft tumors and tumor weight at endpoint from the indicated groups (LV-oe-AARS1 or LV-sh-AARS1 versus controls, treated with eltrombopag or DMSO as indicated). (b) Tumor growth curves (tumor volume) over time. (c) Representative images of mice from each group. (d) Body weight monitoring during the experiment. (e) Representative immunohistochemistry (IHC) staining of xenograft tissues for HK2, Ki67, N-cadherin, and E-cadherin with quantification (IOD/area). (f) Representative lung images/sections from an experimental metastasis model, with quantification of lung metastatic nodules and tumor occupancy of lung volume. Each dot represents one mouse; data are shown as mean ± SD; P values and “ns” are indicated.
